# Crystalline functionalized endohedral C_60_ metallofullerides

**DOI:** 10.1038/s41467-018-05496-8

**Published:** 2018-08-06

**Authors:** Ayano Nakagawa, Makiko Nishino, Hiroyuki Niwa, Katsuma Ishino, Zhiyong Wang, Haruka Omachi, Ko Furukawa, Takahisa Yamaguchi, Tatsuhisa Kato, Shunji Bandow, Jeremy Rio, Chris Ewels, Shinobu Aoyagi, Hisanori Shinohara

**Affiliations:** 1 Department of Chemistry and Institute for Advanced Research, Nagoya University, Nagoya, 464-8602 Japan; 2 Center for Coordination of Research Facilities, Institute for Research Promotion, Niigata University, Niigata, 950-2181 Japan; 3 Graduate School of Human and Environmental Sciences, Kyoto University, Sakyo-ku, Kyoto, 606-8501 Japan; 4 Faculty of Science and Technology, Department of Applied Chemistry, Meijo University, Nagoya, 468-8502 Japan; 5 Institut des Materiaux Jean Rouxel (IMN), Université de Nantes, CNRS UMR6502, BP32229, 44322 Nantes, France; 6 Department of Information and Basic Science, Nagoya City University, Nagoya, 467-8501 Japan

## Abstract

Endohedral metallofullerenes have been extensively studied since the first experimental observation of La@C_60_ in a laser-vaporized supersonic beam in 1985. However, most of these studies have focused on metallofullerenes larger than C_60_ such as (metal)@C_82_, and there are no reported purified C_60_-based monomeric metallofullerenes, except for [Li@C_60_]^+^(SbCl_6_)^−^ salt. Pure (metal)@C_60_ compounds have not been obtained because of their extremely high chemical reactivity. One route to their stabilization is through chemical functionalization. Here we report the isolation, structural determination and electromagnetic properties of functionalized crystalline C_60_-based metallofullerenes Gd@C_60_(CF_3_)_5_ and La@C_60_(CF_3_)_5_. Synchrotron X-ray single-crystal diffraction reveals that La and Gd atoms are indeed encapsulated in the *I*_h_-C_60_ fullerene. The HOMO-LUMO gaps of Gd@C_60_ and La@C_60_ are significantly widened by an order of magnitude with addition of CF_3_ groups. Magnetic measurements show the presence of a weak antiferromagnetic coupling in Gd@C_60_(CF_3_)_3_ crystals at low temperatures.

## Abstract (web summary)

While endohedral metallofullerenes have demonstrated advantageous electronic and magnetic properties, the isolation of monomeric M@C_60_ remains highly challenging. Here, the authors prepare trifluoromethylated Gd@C_60_ and La@C_60_, where the functionalization of C_60_ allows for the stabilization of these otherwise reactive species.

## Introduction

C_60_ fullerene is the most abundant member of the so-called fullerene family^1–4^. However, monomeric C_60_-based endohedral metallofullerenes (i.e., fullerenes with metal atom(s) encapsulated), referred to hereafter as M@C_60_, have not been obtained in macroscopic and pure form to date. Most previous studies on metallofullerenes reported during the past 25 years have focused on higher fullerenes than C_60_ such as C_80_, C_82_, and C_84_^2^, even though the presence of M@C_60_ has been confirmed by mass spectrometry of supersonic cluster beams in the gas phase^3^ as well as laser-vaporized processed soot^4^. Theoretical calculations also suggested the stability and possible structure of M@C_60_^5–7^. An exception is [Li@C_60_]^+^(SbCl_6_)^−^ salt, which was produced by Li-ion bombardment with C_60_ onto a target plate in vacuum^8, 9^. Neutral Li^+^@C_60_^∙−^ radicals have been obtained by the electrochemical reduction of cationic Li^+^@C_60_; however, Li@C_60_ oligomerizes^10^ and has a dimerized form in the crystal^11^.

The inability to extract and purify M@C_60_ is due to their very small highest occupied molecular orbital-lowest unoccupied molecular orbital (HOMO-LUMO) gaps predicted, which lead to high chemical reactivity toward surrounding soot, air, moisture, and various organic solvents. There have been, however, several attempts^12–15^ to try and extract and purify M@C_60_ by, for example, vacuum sublimation from soot followed by liquid chromatographic separation^13, 14^. Unfortunately, none of these efforts led to the final macroscopic preparation of pure and isolated M@C_60_. For this reason, M@C_60_ are often referred to as the missing metallofullerenes^2, 16, 17^.

One of the main reasons for the long-standing interest in M@C_60_ is due to their expected novel electronic and magnetic behavior at low temperatures such as superconductivity, because the electronic band structures near the Fermi levels of M^3+^@C_60_^3−^ solids resemble those of superconducting alkali-doped C_60_ fullerides such as (K_3_)^3+^C_60_^3− ^^18^. If this is the case, the presence of heavy atoms like lanthanoid elements inside C_60_ might enhance the superconducting transition temperatures^19^. One way to stabilize such species and open the HOMO-LUMO gap is through chemical functionalization. Here we report the macroscopic preparation of purified (>99.9%) trifluoromethylated Gd@C_60_ and La@C_60_, stable as Gd@C_60_(CF_3_)_3,5_ and La@C_60_(CF_3_)_3,5_, respectively, and their structural determination, and electronic and magnetic properties.

## Results

### Enhanced stabilization of Gd@C_60_ and La@C_60_

To stabilize Gd@C_60_ and La@C_60_, we have employed in situ trifluoromethylation during the arc-discharge synthesis of the metallofullerenes developed in the present laboratory (see Supplementary Discussion 1 and Supplementary Figure 1)^11, 19^. For example, the in situ trifluoromethylation of Gd@C_60_ generates Gd@C_60_(CF_3_)_*n*_ (*n* = 1–6) together with other sizes of trifluoromethylated Gd-metallofullerenes, Gd@C_2*m*_(CF_3_)_*n*_ (2*m* ≥ 70) (see Supplementary Figures 2 and 3 for La). The high-temperature arc-discharge induces evaporation of polytetrafluoroethylene (PTFE) placed near the arc zone to produce CF_3_ radicals^11^. We found trifluoromethyl-derivatized metallofullerenes, Gd@C_60_(CF_3_)_*n*_ and La@C_60_(CF_3_)_*n*_ (*n* = 3, 5), were formed efficiently. Trifluoromethyl derivatives of Gd@C_60_ and La@C_60_ are totally soluble and stable in toluene and carbon disulfide, which enabled us to perform high-performance liquid chromatography (HPLC) purification and thus subsequent characterization. As discussed in later sections, the stability of these trifluoromethyl derivatives, Gd@C_60_(CF_3_)_3,5_ and La@C_60_(CF_3_)_3,5_, can be attributed to their closed-shell electronic structures, leading to wider HOMO-LUMO gaps than those of pristine Gd@C_60_ and La@C_60_, respectively. With the current separation/isolation protocol (see Supplementary Figure 4), one may, for example, obtain ca. 1.0–2.0 mg of purified (>99.9%) Gd@C_60_(CF_3_)_5_ within 24 h from the initial *o*-xylene extraction of the soot containing Gd@C_2*m*_(CF_3_)_*n*_ obtained by 10 arc-discharge syntheses (see Supplementary Figures 5–8). The absorption onsets of the ultraviolet-visible-near-infrared (UV-Vis-NIR) absorption spectra of CS_2_ solution of Gd@C_60_(CF_3_)_5_ and La@C_60_(CF_3_)_5_ suggest the presence of enlarged HOMO-LUMO gaps of ca. 1.2 eV approaching that of C_60_ (1.6 eV) (see Supplementary Figure 9). Interestingly, Gd@C_60_(CF_3_) and Gd@C_60_(CF_3_)_2_ have not been solvent-extracted because of their much smaller HOMO-LUMO gaps even though mass spectrometric analysis indicates the presence of these metallofullerenes in raw soot.

Because of the very high reactivity of M@C_60_, it has not been self-evident that the metal is encapsulated in the conventional truncated icosahedral *I*_h_-C_60_. The C_60_ might have a so-called non-isolated pentagon rule (non-IPR) structure^20, 21^, where two (or three) pentagons are fused with each other, since non-IPR fullerenes have frequently been observed in metallofullerenes during the past decade^2^. To answer this question, the molecular and crystal structures of Gd@C_60_(CF_3_)_5_ (structural isomers I and II) and La@C_60_(CF_3_)_5_ (I) have been determined by synchrotron radiation single-crystal X-ray diffraction (XRD) at SPring-8 (see Supplementary Tables 1–5).

### Synchrotron single-crystal XRD measurements

**Fig. 1:**
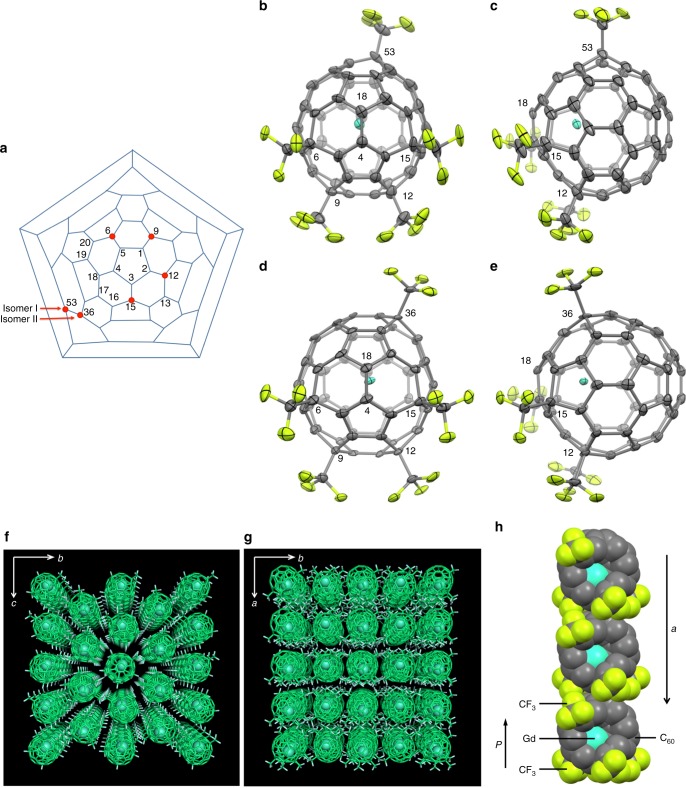
Gd@C_60_(CF_3_)_5_ (I) and (II). **a** Schlegel diagram of C_60_. CF_3_ groups attached to Gd@C_60_(CF_3_)_5_ (I) and (II) are shown as red circles. **b**, **c** Molecular structure of Gd@C_60_(CF_3_)_5_ (I). **d**, **e** Molecular structure of Gd@C_60_(CF_3_)_5_ (II). Front views facing the bond between carbon atoms 4 and 18 (**b**, **d**) and side views (**c**, **e**) are shown. The thermal ellipsoids in (**b**–**e**) are drawn at 50% probability level. **f**, **g** Molecular packing in the Gd@C_60_(CF_3_)_5_ (I) crystal viewed along the *a*- and *c*-axis, respectively. Pseudo-hexagonal close-packed layers in the *bc*-plane are stacked along the *a*-axis. **h** Steric and electrostatic stacking of the Gd@C_60_(CF_3_)_5_ (I) molecules with an electric dipole moment (*P*) along the *a*-axis, which form a close-packed array

The X-ray results clearly show that the C_60_ cage structures of Gd@C_60_(CF_3_)_5_ (I, II) and La@C_60_(CF_3_)_5_ (I) are very similar to that of the conventional empty C_60_(*I*_h_). The five CF_3_ groups of isomer I are attached to carbon atoms numbered as 6, 9, 12, 15, and 53 in a Schlegel diagram shown in Fig. 1a. In isomer II the five CF_3_ groups are attached to carbon atoms numbered as 6, 9, 12, 15, and 36. In both isomers, four of the five CF_3_ groups are attached to carbon atoms 6, 9, 12, and 15 bonded to carbon atoms 5, 1, 2, and 3 on a pentagon. Figure 1b–e shows the molecular structures of Gd@C_60_(CF_3_)_5_(I) and (II), respectively. Very interestingly, the remaining CF_3_ group is attached not to carbon atom 18 but to carbon atom 53 (isomer I) or 36 (isomer II) located far from the 1-2-3-4-5 pentagon. This asymmetric attachment of five CF_3_ groups results in *C*_1_ symmetry. If the fifth CF_3_ group had been attached to carbon atom 18, neighbouring carbon atom 4 on the pentagon, the molecule would have had five-fold symmetry. Instead of the fifth CF_3_ group, a Gd (La) atom inside the C_60_ cage is located near carbon atoms 18 and 4. The distances between the Gd atom and carbon atoms 18 and 4 are ~2.38 Å in both isomers (see Supplementary Tables 2–5).

The selective location of the Gd (La) atom is explained in terms of the elongation of C–C bond lengths by the attachment of CF_3_ groups. The conventional C_60_(*I*_h_) molecule consists of two kinds of C–C bonds: short 6:6 bonds fusing two hexagons with a bond length of 1.39 Å and longer 6:5 bonds fusing a hexagon and a pentagon with a bond length of 1.45 Å. As expected, the C–C bonds around the carbon atoms with CF_3_ groups attached (6, 9, 12, 15, and either 53 or 36) have *sp*^3^ character, resulting in the elongation of the bonds. The 6:6 bond lengths between 6-5, 9-1, 12-2, and 15-3 carbon atoms are ~1.51 Å in both of the isomers (see Supplementary Tables 2 and 5). The 6:6 bond lengths between 18 and 4 atoms (~1.46 Å) are also longer than the normal 6:6 bond length (1.39 Å). This suggests that the Gd is interacting with the *p*_*z*_-orbitals of atoms 18 and 4, resulting in the breaking of the *π*-bond and elongation of the bond length. At the same time, there is a general attraction for the Gd toward the area of the C_60_ surface that has been functionalized, hence this location beneath two carbon atoms rather than staying at the center of the neighboring hexagon.

Gd@C_60_(CF_3_)_5_ (I) and La@C_60_(CF_3_)_5_ (I) form pure crystals that contain neither solvent nor ligand molecules. Figure 1f, g shows the molecular packing of the Gd@C_60_(CF_3_)_5_ (I) crystal. The centrosymmetric crystal with a space group of *P*2_1_/*c* contains two chiral isomers of Gd@C_60_(CF_3_)_5_ (I) with the same number of molecules. Most of the endohedral metallofullerene crystals so far reported contain either solvent or ligand molecules^2^. Gd@C_60_(CF_3_)_5_ (II) has been obtained only as a co-crystal with Ni(OEP) (OEP: octaethylporphyrin). The crystals of Gd@C_60_(CF_3_)_5_ (I) and La@C_60_(CF_3_)_5_ (I) are the first example of pure crystals of M@C_60_ derivative. The crystal structure determined possesses a pseudo-hexagonal close-packed layer of the molecules in the *bc*-plane (Fig. 1f) and stacking of the layers along the *a*-axis (Fig. 1g). The stacking of the molecules is caused by intermolecular steric and electrostatic interactions. The molecule has an electric dipole moment (*P*) because of the electron-withdrawing CF_3_ groups asymmetrically attached to the C_60_ cage and the presence of the off-centered Gd (La) atom inside. The stacking of the turtle-like molecules with an electric dipole moment can provide an electrostatically stable close-packed allay along the *a*-axis as shown in Fig. 1h (see Supplementary Figures 10–11). The detailed crystallographic data are summarized in Supplementary Tables 1–6.

We then carried out ^19^F-nuclear magnetic resonance (NMR) measurements of La@C_60_(CF_3_)_n_ (see Supplementary Discussion 3 and Supplementary Figure 14). The spectra of the two La@C_60_(CF_3_)_5_ isomers consist of five equal-intensity peaks, which are four quartets (or multiplets) due to the through-space coupling of the four grouped CF_3_ units and the other singlet due to fifth more isolated CF_3_ unit^22, 23^. In contrast, the ^19^F-NMR spectrum of La@C_60_(CF_3_)_3_ only showed a singlet peak at −75.5 ppm because the three trifluoromethyl groups are geometrically equivalent, indicating *C*_3_ symmetry of La@C_60_(CF_3_)_3_.

## Discussion

To obtain information on the growth mechanism, structure and electronic properties of Gd@C_60_(CF_3_)_5_, we perform spin unrestricted density functional calculations under the local spin density approximation^24–26^, applying relativistic pseudopotentials^27^. Wavefunctions are constructed from 38/90/28 independent Gaussian-based functions for C/Gd/F, respectively, up to *l* = 3 angular momentum, with an electron temperature of *k*_B_*T* = 0.04 eV for level occupation and 300 Ha cut-off plane-waves for the charge density. To explore the addition pathways for CF_3_ functionalization of Gd@C_60_(CF_3_)_*n*_, *n* = 0-5, we assume that CF_3_ adds to the most thermodynamically favored site and is then immobile, which is justified by a calculated high surface migration barrier of CF_3_ (*n* = 1) of 59 kcal mol^−1^. Taking the most stable *n*th isomer as a starting point for *n* + 1 stepwise addition allows us to reduce the number of isomers considered to a manageable several hundreds. Where two stable isomers are closer in energy than 2.3 kcal/mol of each other, we have used both as starting points for subsequent addition, allowing us to trace bifurcations in the addition pathway.

**Fig. 2:**
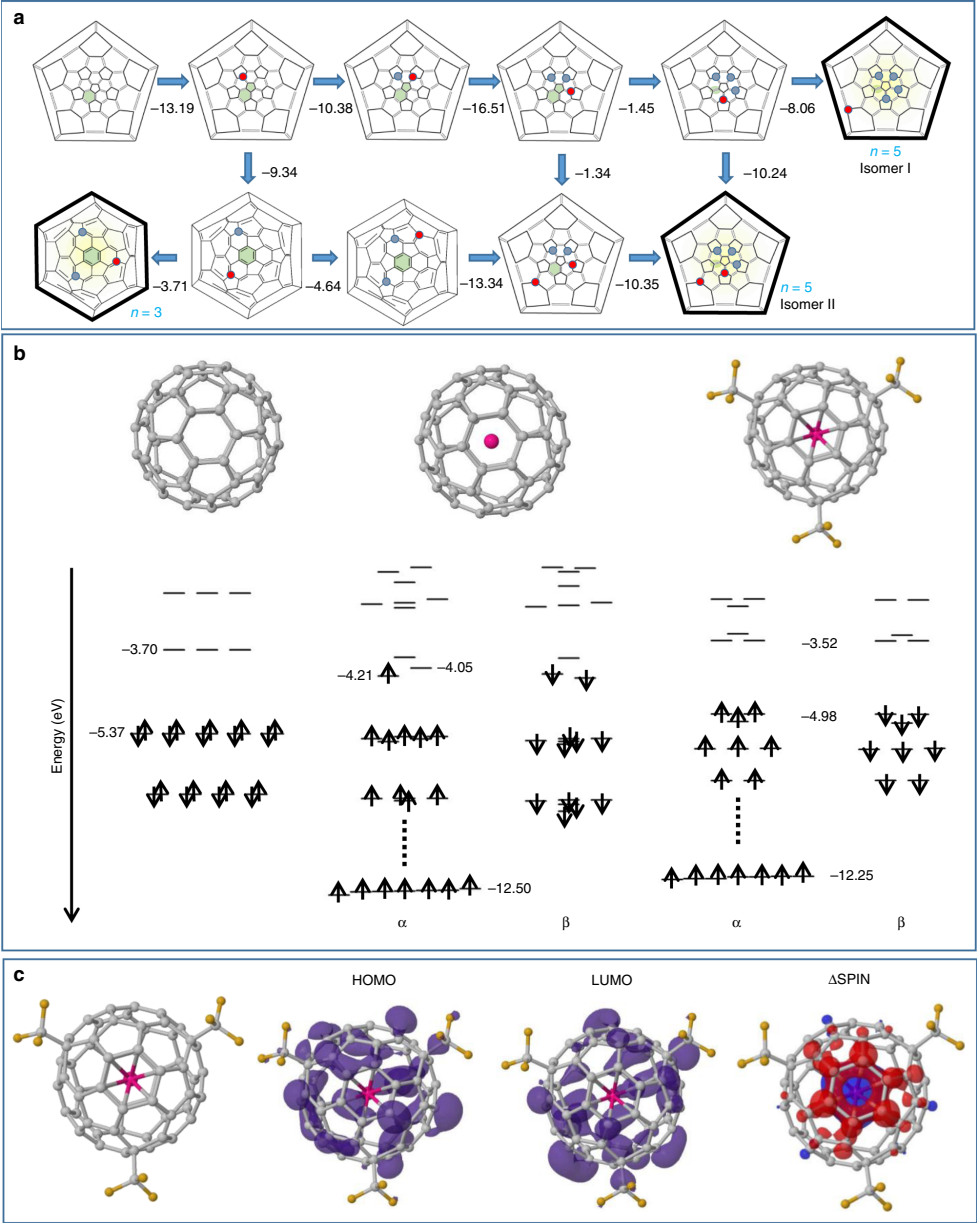
Calculated functionalization sequences and electronic properties of Gd@C_60_(CF_3_)_*n*_. **a** Calculated preferential addition sequences for CF_3_ to Gd@C_60_. Previous addition sites marked in blue, new addition site in red. Pink shading indicates location of the encapsulated Gd atom. Numbers indicate energy release in kcal mol^−1^ according to the reaction Gd@C_60_(CF_3_)_*n* − 1_ + ½ (CF_3_)_2_ → Gd@C_60_(CF_3_)_*n*_. All pathways are shown where isomers are within 2.3 kcal mol^−1^ of the most stable. Fullerenes where next addition is endothermic have black outlines and represent stable end points of a reaction pathway. **b** Calculated Kohn-Sham eigenvalues (eV) for (left) C_60_, (center) Gd@C_60_, and (right) Gd@C_60_(CF_3_)_3_, showing CF_3_ functionalization results in a closed-shell system, reopening the HOMO-LUMO gap to nearly that of C_60_. **c** Spatial distribution of calculated Kohn-Sham eigenstates showing highest occupied molecular orbital (HOMO) and lowest unoccupied molecular orbital (LUMO) and their neighboring states around the Fermi level for the most stable Gd@C_60_(CF_3_)_5_(I) isomer. The states are delocalized across the fullerene cage with very limited CF_3_ character

The Gd atom in Gd@C_60_ is initially covalently bound beneath hexagon (3-4-15-16-17-18) with 2.41 Å Gd–C bond lengths. The most stable CF_3_ addition pathway involves sequential addition to back bonds of the 1-2-3-4-5 pentagon, i.e., addition at sites 6, 9, 12, and 15 in that order shown in top line of Fig. 2a. Instead of completing the pentagon at site 18, which is excluded due to the presence of Gd below this last pentagon back bond with ~2.35 Å Gd–C bond lengths, the fifth CF_3_ group adds behind the neighboring pentagon at sites 53 (isomer I) or 36 (isomer II). Adding a sixth CF_3_ group is endothermic compared to ½(CF_3_)_2_, confirming that *n* = 5 is the end point in the addition sequence in agreement with the experimental observation. Interestingly, isomer II matches a La@C_60_(CF_3_)_5_ structure proposed previously^7^, but the intermediate *n* = 4 structure proposed there is different to the structure we find here and can only be linked to *n* = 3 and 5 with additional surface CF_3_ migration.

After *n* = 1 addition at site 6 there is a second stable isomer for *n* = 2, where Gd maintains its hexagonal site and CF_3_ adds to site 36 (lower line of Fig. 2a). The pathway then bifurcates once again, the energetically favored route continuing the 9, 12, 15 addition sequence to isomer II. However, an alternative *n* = 3 addition at site 13 results instead in a *C*_3_ symmetry Gd@C_60_(CF_3_)_3_ isomer. Further CF_3_ addition to this is endothermic, showing this to be a third stable end point in the addition sequence. This mapping process thus shows remarkable agreement with the current X-ray and ^19^F-NMR experimental results, successfully predicting the three observed Gd@C_60_(CF_3_)_n_ species, i.e., Gd@C_60_(CF_3_)_5_ (I), Gd@C_60_(CF_3_)_5_ (II) and Gd@C_60_(CF_3_)_3_.

These three stable isomers have multiplicity 7/2, associated with the Gd atom. They are closed-shell systems (unlike many of the less-stable isomers), restoring the gap between HOMO and LUMO to ~2/3 that of C_60_ (Fig. 2b) with the largest gap for Gd@C_60_(CF_3_)_3_. The unpaired Gd *f*-states are deep lying with the HOMO and LUMO localized primarily over the fullerene cage with minor CF_3_ character (Fig. 2c). Gd plays an interesting dual role when selecting CF_3_ addition sites, acting as donor and localizing surface charge, while also affecting surface chemistry through strong hybridization with the carbon 2*p* states. This suggests different addition sequences are likely for non-covalently bound endohedral +3 oxidation species. As seen above, the agreement between the X-ray and the theoretical results for the presence of the stable isomers of Gd@C_60_(CF_3_)_3,5_ strongly suggests that CF_3_ groups are sequentially added to Gd@C_60_ during the arc-discharge synthesis. The energy levels, HOMO-LUMO and spin-up and -down charge density for Gd@C_60_(CF_3_)_3_ and Gd@C_60_(CF_3_)_5_ (I,II) are shown in Supplementary Figures 15 and 16, respectively.

Literature calculations for M@C_60_(CF_3_)_3_ using comparable methods give a calculated HOMO-LUMO gap of 0.81 eV for Y@C_60_(CF_3_)_3_^16^, and ~0.7 eV for La@C_60_(CF_3_)_3_^7^, at first sight in discrepancy with our significantly larger calculated gap for Gd@C_60_(CF_3_)_3_ of 1.4 eV. This is because the literature calculations consider a M@C_60_(CF_3_)_3_ isomer with the CF_3_ groups arranged around a shared pentagon. Our calculated HOMO-LUMO gap for this Gd@C_60_(CF_3_)_3_ isomer is indeed 0.78 eV, close to these values. However, we do not predict this structure to be stable in isolation but simply an intermediate structure en-route to the formation of isomer I and isomer II Gd@C_60_(CF_3_)_5_ structures. The Gd@C_60_(CF_3_)_3_ isomer we predict to be a stable end point is instead the *C*_3_ symmetry isomer shown in Fig. 2, with CF_3_ groups arranged symmetrically around a central hexagon. This arrangement of CF_3_ groups gives the much wider calculated HOMO-LUMO gap of 1.4 eV.

**Fig. 3:**
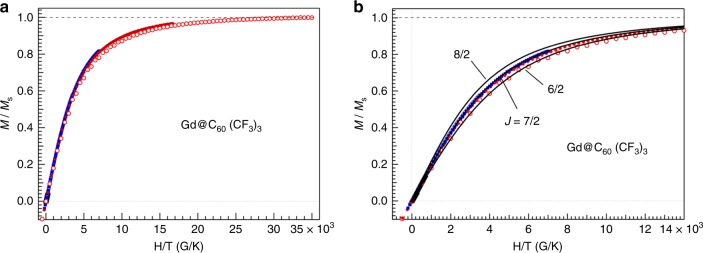
Normalized magnetization curves of Gd@C_60_(CF_3_)_3_. **a** Whole trace of magnetization taken at 273, 100, 20, 10, 4.2, and 2 K, and **b** is an expansion around the origin of abscissa. *M*_s_ value determined experimentally is 41108 emu · G mol^−1^. Solid lines are the calculated magnetization curves using Brillouin function with *J* = 8/2, 7/2, and 6/2. Red open circles are for the data taken at 2 K. Only this data set did not fit with the same curvature

The magnetic properties of Gd@C_60_(CF_3_)_3_ metallofullerene was investigated by SQUID measurements (see Supplementary Discussion 5). The HOMO-LUMO gap of Gd@C_60_(CF_3_)_3_ (1.4 eV) is larger than that of Gd@C_60_(CF_3_)_5_ (1.2 eV) suitable for solvent extraction and HPLC isolation. Magnetization curves of solid Gd@C_60_(CF_3_)_3_ indicate no hysteresis as shown in Fig. 3, where the ordinate is normalized by experimentally determined saturation magnetization *M*_s_ of 41 108 emu ∙ G mol^−1^. Except for the magnetization curve at 2 K, other data points are on the same curvature trace. When magnetic moments thermally fluctuate without strong interaction between the magnetic moments, magnetization curves are fitted by the following Brillouin function *B*_*J*_(*x*)

1 $$M = M_{\mathrm{s}}B_J\left( x \right)$$

2 $$M_{\mathrm{S}} = NgJ\mu _{\mathrm{B}}$$

3 $$B_J\left( x \right) = \frac{{2J + 1}}{{2J}}{\mathrm{coth}}\left( {\frac{{2J + 1}}{{2J}}x} \right) - \frac{1}{{2J}}{\mathrm{coth}}\left( {\frac{1}{{2J}}x} \right)$$

4 $$x = \frac{{gJ\mu _{\mathrm{B}}H}}{{k_{\mathrm{B}}T}}$$

where *N* is the spin concentration, *g* the *g*-factor, *J* the total quantum number, *μ*_B_ the Bohr magneton, and *k*_B_ the Boltzmann constant. From the curve fitting, we found that the total quantum number *J* = 7/2 is needed to explain the experimental results by setting *g* = 2. This gives an effective magnetic moment *μ*_eff_ $\left( { = g\sqrt {J\left( {J + 1} \right)} \mu _{\mathrm{B}}} \right)$ of 7.94 *μ*_B_ per Gd@C_60_(CF_3_)_3_ molecule. This experimental analysis supports a trivalent Gd^3+^ state by transferring three electrons to the C_60_ cage (Gd^3+^@C_60_^3−^) and these electrons are then used to make chemical bonds with three CF_3_ on C_60_. Mulliken charge analysis shows that most of the transferred charge is indeed spread around the C_60_ cage beneath the CF_3_ groups, on the central pentagon, and on the neighbors of the Gd. The magnetic moment is thus essentially located on the encaged Gd^3+^ i on (4*f*^7^, *J* = 7/2 with orbital quantum number *L* = 0, i.e., Gd^3+^ has spin 7 *μ*_B_) in agreement with the theoretical result shown in Fig. 2b. According to the previous studies on M^3+^@C_82_^3−^ (M = Gd, Dy, Ho, Er) metallofullerenes^28–32^, the magnetic moments observed are significantly small compared to the expected moments of free M^3+^ ions.

This reduction of the magnetic moments was theoretically explained by antiferromagnetic coupling between the trivalent endohedral metals and free spins on the carbon cage^33^. In the present Gd (La)-encapsulating C_60_ fullerides, since free spins on C_60_ are very small by forming the chemical bonds with CF_3_ on the cage, this guarantees an almost ideal magnetic moment of Gd^3+^ ion. Theoretically, a trace counter-spin can be seen localized on the C_60_ cage between the Gd and CF_3_ groups at 0 K (Fig. 2c), presumably too weak to be detectable by SQUID but partially responsible for the weak antiferromagnetic coupling observed at low temperature. This was also confirmed by the analysis of temperature dependence of molar magnetic susceptibility as explained in Supplementary Discussion 5.

**Fig. 4:**
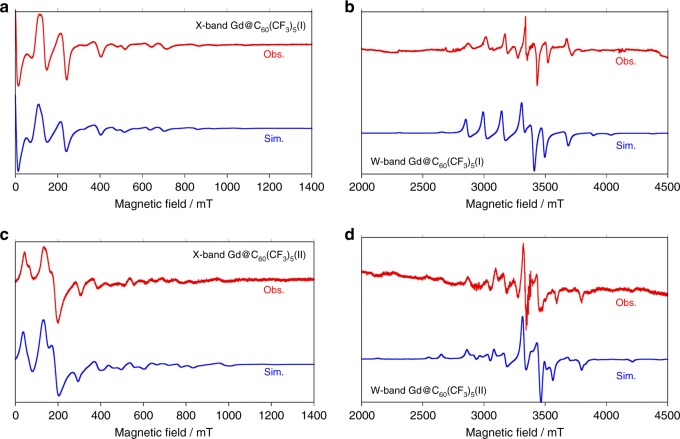
Confirmation of ESR parameters for Gd@C_60_(CF_3_)_5_(I) and Gd@C_60_(CF_3_)_5_(II). **a**, **c** ESR spectra (red lines) of Gd@C_60_(CF_3_)_5_(I) and Gd@C_60_(CF_3_)_5_(II) recorded by X-band (9 GHz) spectrometer in CS_2_ at 4 K, **b**, **d** W-band (90 GHz) spectrometer in CS_2_ at 20 K. ESR parameters were determined so that both observed spectra in **a**–**d** should be simultaneously reproduced by simulation (blue lines)

To further investigate the electronic structures of Gd@C_60_(CF_3_)_5_ (I,II), we also performed electron spin resonance (ESR) measurements in CS_2_ frozen solution (see Supplementary Discussion 6 and Supplementary Table 7). The spectra recorded by an X-band continuous wave ESR spectrometer at 4 K and by a W-band at 20 K are shown in the upper and lower panels of Fig. 4, respectively, where the observed spectra with fine structure in red are compared with simulated ones in blue^34, 35^. The parameters shown in Supplementary Table 7 were determined so that both of the observed spectra by the X- and W-band measurements should simultaneously be reproduced by the simulation. In both cases the spin quantum number of *S* = 7/2 was obtained, consistent with the theoretical and magnetic measurement results described above. The zero-field splitting parameters, *D* = 0.162 cm^−1^ and *D* = 0.193 cm^−1^, were obtained for Gd@C_60_(CF_3_)_5_ (I) and Gd@C_60_(CF_3_)_5_ (II), respectively, comparable to those for *S* = 7/2 states of Gd@C_82_^34^ and Eu@C_82_^35^. The lift of the *x* and *y* degeneracy of *E* = 0.012 cm^−1^ for Gd@C_60_(CF_3_)_5_ (II), which is bigger than that (*E* = 0.000 cm^−1^) of Gd@C_60_(CF_3_)_5_ (I), suggests that the position of the Gd^3+^ ions in the two isomers differs slightly, since the *E* parameters originate basically in the spin-orbit coupling of the Gd^3+^ ions. Our calculations indeed indicate that the Gd^3+^ ion of isomer (I) lies more symmetrically beneath the C–C bond (so more symmetrically with respect to the four CF_3_ groups around the pentagon) than that of isomer (II). The observed ESR parameters are summarized in Supplementary Table 7.

In summary, the missing metallofullerenes, Gd@C_60_ and La@C_60_, in the trifluoromethylated forms of Gd@C_60_(CF_3_)_3_,_5_ and La@C_60_(CF_3_)_5_, have been obtained in pure bulk forms. In situ trifluoromethylation widens the HOMO-LUMO gaps of Gd@C_60_ and La@C_60_, enabling isolation of the metallofullerenes and the subsequent single-crystal synchrotron XRD. The crystals exhibit a pseudo-hexagonal close-packed layer of molecules in the *bc*-plane and stacking of the layers along the *a*-axis (a turtle-like stacked structure). The temperature dependence of the magnetic susceptibility of Gd@C_60_(CF_3_)_3_ is well fitted by the Curie-Weiss law. The magnetic moment is located on the encaged Gd^3+^ i on (4*f*^7^, *J* = 7/2 with orbital quantum number *L* = 0), with very small magnetic moment on the cage in agreement with the theoretical results. The intact forms of Gd@C_60_ and La@C_60_ might exhibit superconductivity as the electronic structures resemble those of superconducting alkali-doped C_60_ fullerides. The preparation and isolation of intact Gd@C_60_ fulleride is now underway in the present laboratory.

## Methods

### Synthesis, purification, and crystallization

A cross-sectional view of the DC arc-discharge chamber is illustrated in Supplementary Figure 1, where PTFE rods (40 g) are placed near the discharge area. Graphite rods (100 g) impregnated with Gd (La) (0.8 mol%, Toyo Tanso Co.LTD) was used as the anode. A pure graphite rod (Toyo Tanso Co.LTD) was used as the cathode. Arc-discharge was performed at a DC current of 500 A in a flowing He atmosphere with a pressure of 7–9 kPa. During arc-discharge because of the high-temperature around the arc zone, PTFE was decomposed and evaporated to produce CF_3_ radicals. Normally, 50–70 g of raw soot was obtained per discharge. Gd-metallofullerenes and empty fullerenes were extracted from the raw soot with *o*-xylene.

### Extraction and separation

The rapid separation of the metallofullerenes from empty fullerenes was carried out by the TiCl_4_ Lewis acid method developed in the present laboratory. To a 500 mL CS_2_ solution of the crude mixture of Gd-fullerenes and empty fullerenes, ca. 5 mL of TiCl_4_ was added. Metallofullerenes were reacted immediately and insoluble complexes were precipitated out. After mixing for 5 min, the precipitate was collected on a PTFE membrane filter and washed with 10–20 mL of CS_2_ to separate from the empty fullerenes solution. Deionized water was passed through the filter to decompose the complex of metallofullerene/TiCl_4_, and then washed with acetone to eliminate extra water. Finally, CS_2_ was passed through the filter to collect desired Gd-metallofullerenes as a solution as shown in Supplementary Figure 2 (equivalent for La in Supplementary Figure 3).

### Multi-stage HPLC purification

HPLC purification was conducted by using a JAI (Japan Analytical Industry Co. LTD.) recycling preparative HPLC LC-9104HS. The overall separation and isolation scheme of Gd@C_60_(CF_3_)_*n*_ is shown in Supplementary Figure 4. For the identification of metallofullerenes, mass spectrometric analysis was performed on Shimadzu MALDI-TOF-MS Spectrometer. Vis/NIR absorption spectra of metallofullerenes in CS_2_ were recorded by a Jasco V-570 spectrophotometer. Three isomers of Gd@C_60_(CF_3_)_*n*_ were isolated from the mixture by the multi-stage HPLC method developed in the present laboratory. Two kinds of columns were used alternatively with toluene eluent for the isolation, i.e., Buckyprep column (20 mm diameter × 250 mm, Nacalai Tesque Inc.) and Buckyprep-M column (20 mm diameter × 250 mm, Nacalai Tesque Inc.). The initial (the first stage) HPLC purification was performed with Buckyprep-M. Gd@C_60_(CF_3_)_*n*_ (*n* = 3, 5) was obtained in fraction 1. The HPLC chromatograms are shown in Supplementary Figure 5 (equivalent for La in Supplementary Figure 6). Identification and isolation of the metallofullerenes were checked by MALDI mass spectroscopy as shown in Supplementary Figures 7 and 8. UV-Vis-NIR absorption spectra of the metallofullerenes exhibit characteristic features and also provide an estimate on their HOMO-LUMO gaps judging from the absorption onsets. The spectral features between Gd- and La-metallofullerenes are almost the same with each isomer as shown in Supplementary Figure 9.

### Synchrotron single-crystal X-ray structure analysis

Single-crystal XRD measurements of Gd@C_60_(CF_3_)_5_ (I), La@C_60_(CF_3_)_5_ (I), Gd@C_60_(CF_3_)_5_ (II), and Gd@C_60_(CF_3_)_3_ were performed at SPring-8 BL02B1. The crystallographic data and various bond lengths for Gd@C_60_(CF_3_)_5_ (I,II), Gd@C_60_(CF_3_)_3_, and La@C_60_(CF_3_)_5_ (I) are summarized in Supplementary Tables 1-6. The crystal structures for Gd@C_60_(CF_3_)_5_ (I), La@C_60_(CF_3_)_5_ (I), and Gd@C_60_(CF_3_)_5_ (II) are shown in Supplementary Figures 10–12, respectively. The charge density surface of Gd@C_60_(CF_3_)_3_ is shown in Supplementary Figure 13.

Gd@C_60_(CF_3_)_5_ (I) and La@C_60_(CF_3_)_5_ (I): Single crystals of Gd@C_60_(CF_3_)_5_ (I) and La@C_60_(CF_3_)_5_ (I) were obtained from CS_2_ solution by vapor diffusion. Results of the XRD measurement are summarized in Supplementary Table 1. The crystal structures were determined by using *SIR* and *SHELX* with good reliable factors. Gd@C_60_(CF_3_)_5_ (I) and La@C_60_(CF_3_)_5_ (I) have similar crystal structures. The centrosymmetric monoclinic crystals consist of the same number of two chiral isomers of the molecule. The unit cell contains two right-handed isomers and two left-handed isomers as shown in Supplementary Figures 10a and 11a. An independent molecule in the asymmetric unit has a disordered structure in which two chiral isomers overlap with the ratios of 0.8 and 0.2 as shown in Supplementary Figures 10b and 11b. In all, 95 C–C, 15 C–F, and 4 C–Gd (La) bond lengths of the major part of the disordered Gd@C_60_(CF_3_)_5_ (I) and La@C_60_(CF_3_)_5_ (I) are listed in Supplementary Tables 2 and 3, respectively. A total of 30 short 6:6 bonds fusing two hexagons and 60 long 6:5 bonds fusing a hexagon and a pentagon on the C_60_ cage are separately shown. The CIF deposition numbers at the Cambridge Crystallographic Data Centre (CCDC) are 1587428 for Gd@C_60_(CF_3_)_5_ (I) and 1587430 for La@C_60_(CF_3_)_5_ (I).

Gd@C_60_(CF_3_)_5_ (II): Single crystals of Gd@C_60_(CF_3_)_5_ (II) were obtained from CS_2_ solution as co-crystals with Ni(OEP) by vapor diffusion. Results of the XRD measurement are summarized in Supplementary Table 4. The crystal structure was determined by using *SIR* and *SHELX* with a good reliable factor. The centrosymmetric triclinic crystals consist of the same number of two chiral isomers of Gd@C_60_(CF_3_)_5_ (II). The unit cell contains a right-handed isomer, a left-handed isomer, three Ni(OEP), and half toluene molecules as shown in Supplementary Figure 12a. An independent molecule in the asymmetric unit has a disordered structure in which two chiral isomers overlap with the ratios of 0.8 and 0.2 as shown in Supplementary Figure 12b. In all, 95 C–C, 15 C–F, and 4 C–Gd bond lengths of the major part of the disordered Gd@C_60_(CF_3_)_5_ (II) are listed in Supplementary Table 5. A total of 30 short 6:6 bonds fusing two hexagons and 60 long 6:5 bonds fusing a hexagon and a pentagon on the C_60_ cage are separately shown. The CIF deposition number at CCDC is 1587429.

Gd@C_60_(CF_3_)_3_: Single crystals of Gd@C_60_(CF_3_)_3_ were obtained from CS_2_ solution by vapor diffusion. Results of the XRD measurement were summarized in Supplementary Table 6. The tetragonal unit cell can contain 16 Gd@C_60_(CF_3_)_3_ molecules. An expected molecular arrangement was obtained by charge flipping using *Superflip* and maximum entropy method using *ENIGMA* as shown in Supplementary Figure 13. The figure shows an electron charge density surface obtained by maximum entropy method. A structure model with 16 double uniform shells of Gd@C_60_ located at (1/4 ± 1/8, 1/4 ± 1/8, 0), (−1/4 ± 1/8, −1/4 ± 1/8, 0), (1/4 ± 1/8, −1/4 ± 1/8, 1/2), and (−1/4 ± 1/8, 1/4 ± 1/8, 1/2) was used in the analysis. Detailed structure of Gd@C_60_(CF_3_)_3_ could not be determined due to a severe orientation disorder and lack of resolution. We also attempted to obtain co-crystals of Gd@C_60_(CF_3_)_3_ with Ni(OEP); however, that have not been obtained.

### 19F-NMR measurements

NMR spectra were recorded on a JEOL ECS-400 spectrometer; chemical shifts for ^19^F-NMR (376 MHz, CDCl_3_/CS_2_) are expressed in parts per million (ppm) relative to C_6_F_6_ (*δ* = –164.9 ppm). Data are reported as follows: chemical shift; multiplicity (s = singlet, q = quartet, m = multiplet); coupling constant (Hz); and integration. La@C_60_(CF_3_)_3_: *δ* –75.5 (s, 9F). La@C_60_(CF_3_)_5_ (I): *δ* –79.4 (s, 3F), –76.0 (q, *J* = 10 Hz, 3F), –74.9 (q, *J* *=* 12 Hz, 3F), –67.4 (m, 3F), and –66.9 (m, 3F). La@C_60_(CF_3_)_5_ (II): *δ* –78.9 (s, 3F), –78.0 (q, *J* *=* 12 Hz, 3F), –73.6 (q, *J* = 10 Hz, 3F), –68.3 (m, 3F), and –67.3 (m, 3F).

### SQUID magnetic measurement

Sample dissolved in CS_2_ was transferred into quartz tube and the solution was vaporized under argon flow. Then the sample in quartz tube was evacuated by using turbo molecular pump at 260 °C for 12 h and vacuum sealed. By this heat treatment, weight of Gd@C_60_(CF_3_)_3_ sample was decreased by ~65% (finally 0.71 mg of sample was loaded in the quartz tube), while no such weight change was observed for La@C_60_(CF_3_)_5_ one. Former finding suggests that the CS_2_ molecules were incorporated in a Gd@C_60_(CF_3_)_3_ solid, forming Gd@C_60_(CF_3_)_3_(CS_2_)_6.4_ just after vaporizing CS_2_ in Ar flow, and such incorporated CS_2_ molecules have been completely removed by heating at 260 °C in vacuo. On the other hand, from the latter fact, La@C_60_(CF_3_)_5_ solid did not incorporate any CS_2_ molecule. That is, all SQUID measurements were carried out by using solution free samples. As described in the Discussion, magnetic behavior can be explained as an intramolecular feature and the interaction between the molecules is very small. Therefore, even if the sample incorporates some CS_2_ molecules, these molecules do not affect the present results.

### ESR measurements

The X- and W-band ESR measurements were performed using a Bruker E500 and a E680 spectrometer, respectively. The temperature was controlled by helium flow cryostat (Oxford Instruments model ITC500). The X-band ESR spectra were measured at 4.0 K, and W-band ESR spectra were measured at 20 K. The spectral simulation were performed by using MATLAB software package with EasySpin toolbox. The observed ESR parameters are shown in Supplementary Table 7.

### Data availability

Structural data are available in Supplementary Information. All additional data such as calculated *xyz* structures are available from the authors on request. The X-ray crystallographic coordinates for structures reported in this study have been deposited at the CCDC, under deposition numbers 1587428–1587430. These data can be obtained free of charge from The CCDC via www.ccdc.cam.ac.uk/data_request/cif.

## Electronic supplementary material

Supplementary Information
