# Supplementary material for: Crystalline functionalized endohedral C60 metallofullerides
Source: Nat Commun. 2018 Aug 6;9:3073. doi: 10.1038/s41467-018-05496-8 (PMC6078994; doi:10.1038/s41467-018-05496-8)
Supplement: Supplementary file 1 — Supplementary Information [file 41467_2018_5496_MOESM1_ESM.pdf]

SUPPLEMENTARY INFORMATION

Crystalline functionalized endohedral C_{60} metallofullerides

Ayano Nakagawa et al.

SUPPLEMENTARY INFORMATION

Crystalline functionalized endohedral C_{60} metallofullerides

Ayano Nakagawa[1], Makiko Nishino[1], Hiroyuki Niwa[1], Katsuma Ishino[1], Zhiyong Wang[1], Haruka Omachi[1], Ko Furukawa[2], Takahisa Yamaguchi[3], Tatsuhisa Kato[3], Shunji Bandow[4], Jeremy Rio[5], Chris Ewels[5], Shinobu Aoyagi[6] & Hisanori Shinohara[1]*

[1]Department of Chemistry and Institute for Advanced Research, Nagoya University, Nagoya 464-8602, Japan.

[2]Center for Coordination of Research Facilities, Institute for Research Promotion, Niigata University, Niigata 950-2181, Japan.

[3]Graduate School of Human and Environmental Sciences, Kyoto University, Sakyo-ku, Kyoto 606-8501, Japan.

[4]Faculty of Science and Technology, Department of Applied Chemistry, Meijo University, Nagoya 478-8502, Japan.

[5]Institut des Materiaux Jean Rouxel (IMN), Université de Nantes, CNRS UMR6502, BP32229, 44322 Nantes, France

[6]Department of Information and Basic Science, Nagoya City University, Nagoya 467-8501, Japan.

Supplementary Discussion 1: Synthesis, purification and crystallization of $Gd@C_{60}(CF_3)_5$ (I), $La@C_{60}(CF_3)_5$ (I) , $Gd@C_{60}(CF_3)_5$ (II) and $Gd@C_{60}(CF_3)_3$

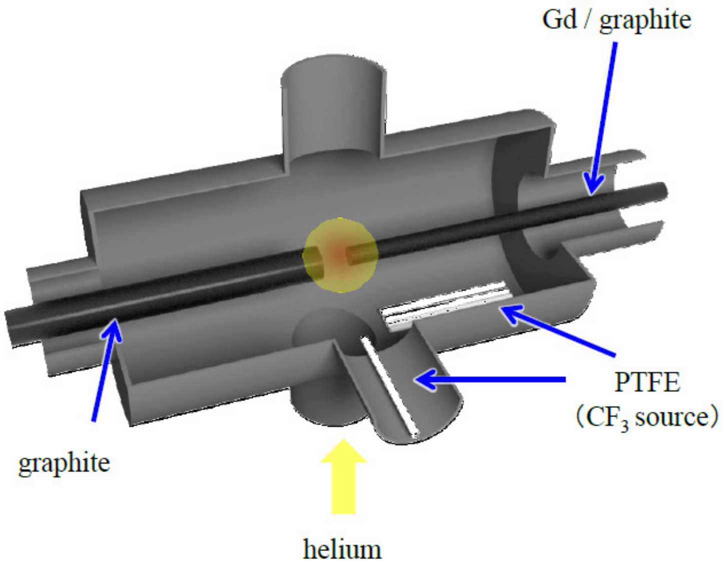

Supplementary Figure 1: A cross-sectional view of the DC arc-discharge apparatus for the synthesis of Gd- metallofullerenes. Polytetrafluoroethene (PTFE) rods are placed near the discharge area for the *in situ* CF_3-functionalization. Image produced by Tsukasa Inoue and used with permission.

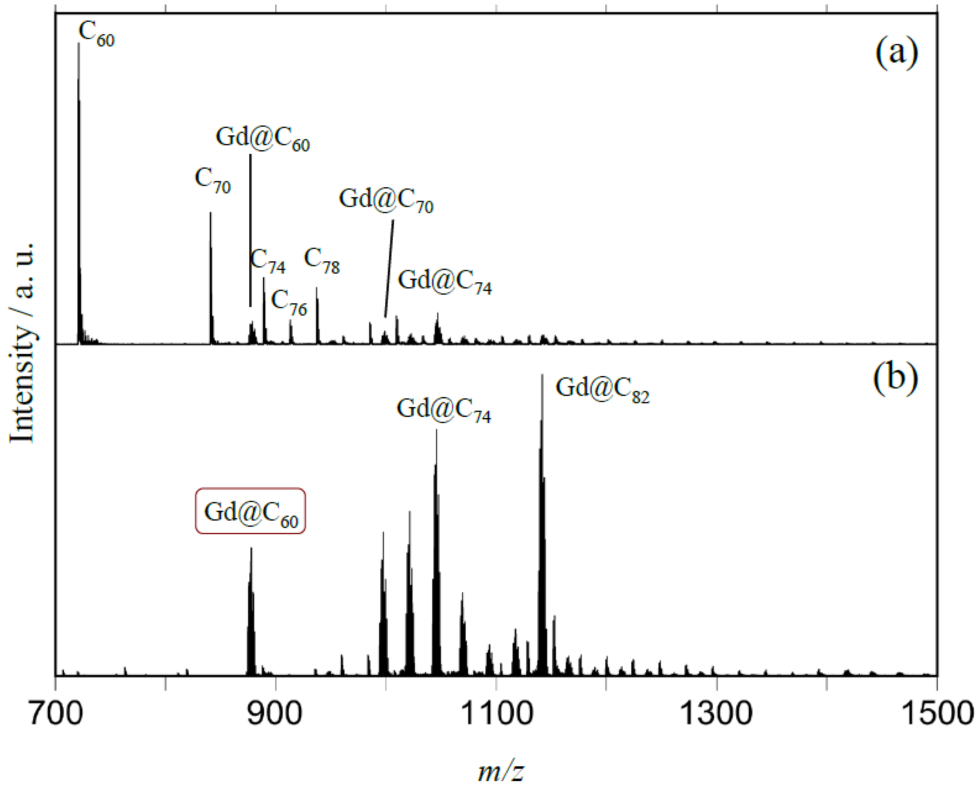

Supplementary Figure 2: a, LD-TOF mass spectra (positive-ion mode, without any matrices) for *o*-xylene extract from raw soot. **b**, LD-TOF mass spectra (positive-ion mode) for separated derivatives of Gd metallofullerenes after the $TiCl_4$ treatment. It should be noted that CF_3- groups are detached from Gd metallofullerenes at the time of laser desorption and ionization, so that only the intact $Gd@C_n$ metallofullerenes are observed (cf. Supplementary Fig. 7).

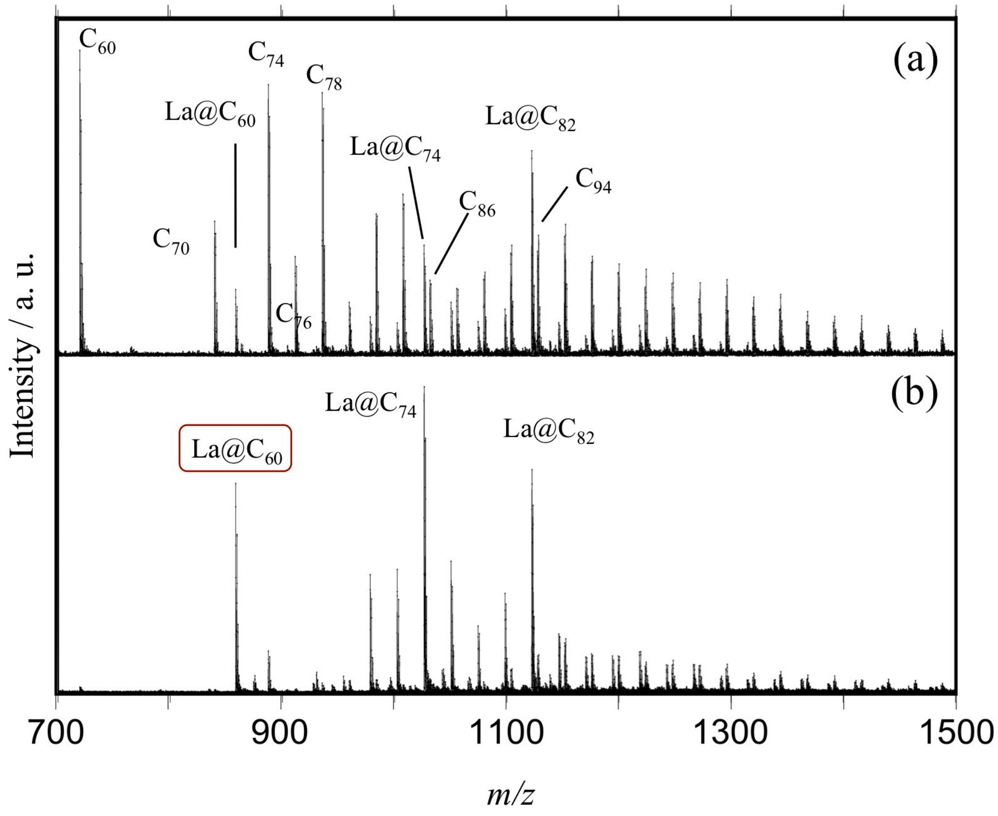

Supplementary Figure 3: a, LD-TOF mass spectra (positive-ion mode, without any matrices) for *o*-xylene extract from raw soot. **b**, LD-TOF mass spectra (positive-ion mode) for separated derivatives of La metallofullerenes after the $TiCl_4$ treatment. It should be noted that CF_3- groups are detached from La metallofullerenes at the time of laser desorption and ionization, so that only the intact La@C_n metallofullerenes are observed (cf. Supplementary Fig. 8).

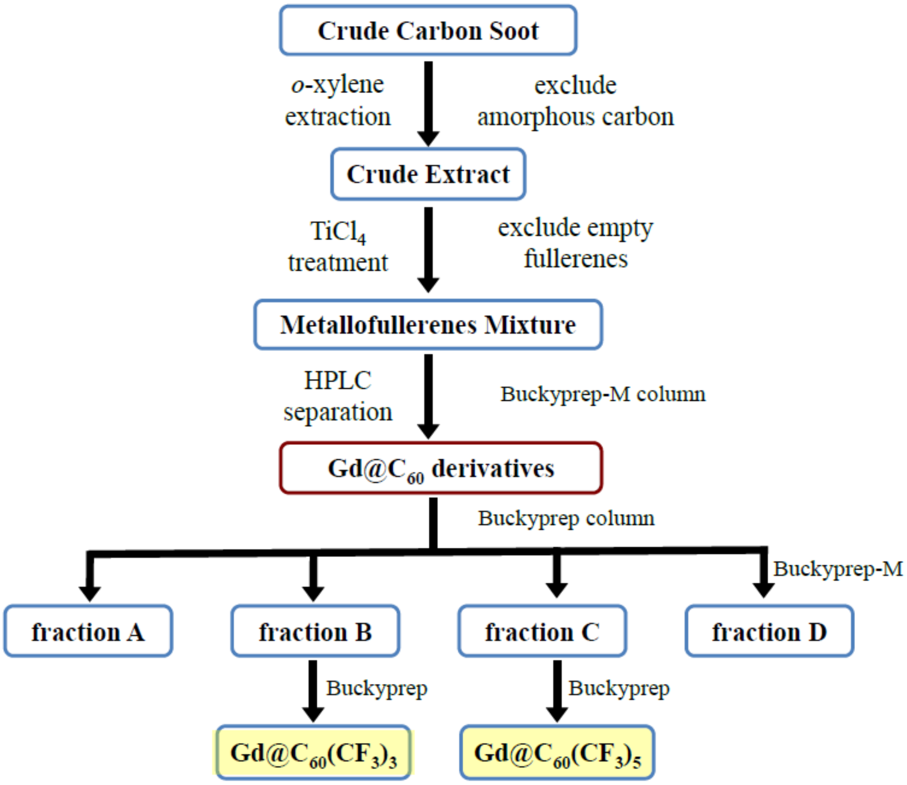

Supplementary Figure 4: The overall separation and isolation scheme of $Gd@C_{60}(CF_3)_5$ and $Gd@C_{60}(CF_3)_3$ from raw soot. The isolation scheme of $La@C_{60}(CF_3)_n$ is almost the same as this.

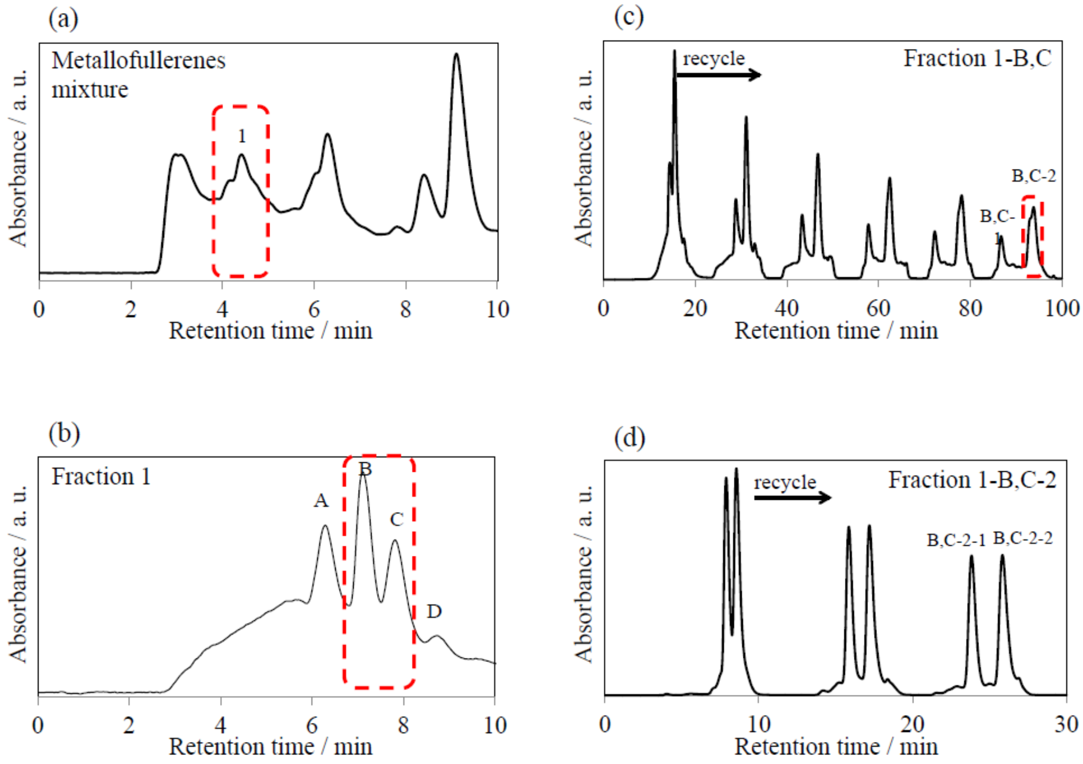

Supplementary Figure 5: a, The first stage HPLC profile (Buckyprep-M column; flow rate: 21 mL/min). The metallofullerenes $Gd@C_{60}(CF_3)_n$ are contained in fraction 1 (3.8-5.2 min). **b**, HPLC profile of fraction 1 (Buckyprep column; flow rate: 16 mL/min). Fractions 1-A,C and D contain isomers of $Gd@C_{60}(CF_3)_5$, whereas fraction 1-B contains $Gd@C_{60}(CF_3)_3$. **c**, HPLC profile of fractions 1-B and 1-C (two Buckyprep-M columns connected in series; flow rate: 12 mL/min). $Gd@C_{60}(CF_3)_5$ (I) is contained in fraction B,C-1. $Gd@C_{60}(CF_3)_3$ and $Gd@C_{60}(CF_3)_5$ (II) are contained in fractions B,C-2. **d**, HPLC profile of fraction 1-B,C-2 (Buckyprep column; flow rate: 16 mL/min). Fractions B,C-2-1 and B,C-2-2 contain $Gd@C_{60}(CF_3)_3$ and $Gd@C_{60}(CF_3)_5$ (II), respectively.

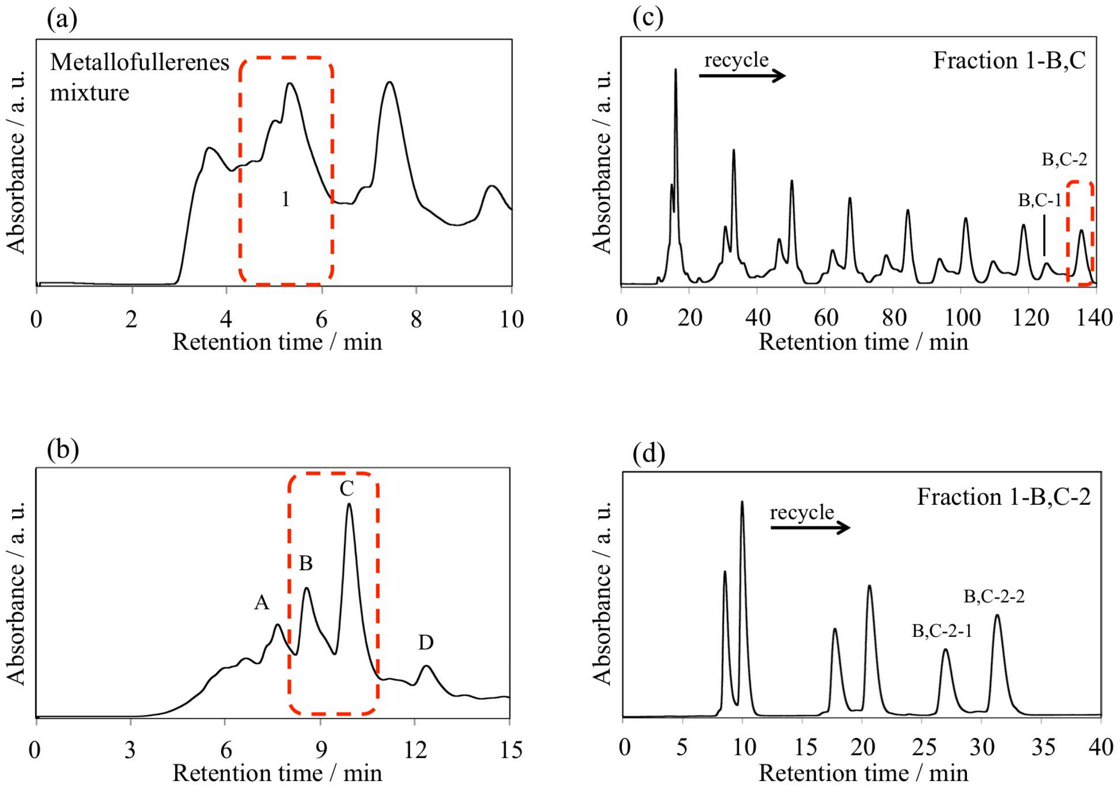

Supplementary Figure 6: **a**, The first stage HPLC profile (Buckyprep-M column; flow rate: 21 mL/min). The metallofullerenes La@$C_{60}(CF_3)_n$ are contained in fraction 1 (4.2-6.2 min). **b**, HPLC profile of fraction 1 (Buckyprep column; flow rate: 16 mL/min). Fractions 1-B,C and D contain isomers of La@$C_{60}(CF_3)_5$, whereas fraction 1-D contains La@$C_{60}(CF_3)_3$. **c**, HPLC profile of fractions 1-B and 1-C (two Buckyprep-M columns connected in series; flow rate: 12 mL/min). La@$C_{60}(CF_3)_5$ (I) is contained in fraction B,C-1. La@$C_{60}(CF_3)_3$ and La@$C_{60}(CF_3)_5$ (II) are contained in fractions B,C-2. **d**, HPLC profile of fraction 1-B,C-2 (Buckyprep column; flow rate: 16 mL/min). Fractions B,C-2-1 and B,C-2-2 contain La@$C_{60}(CF_3)_3$ and La@$C_{60}(CF_3)_5$ (II), respectively.

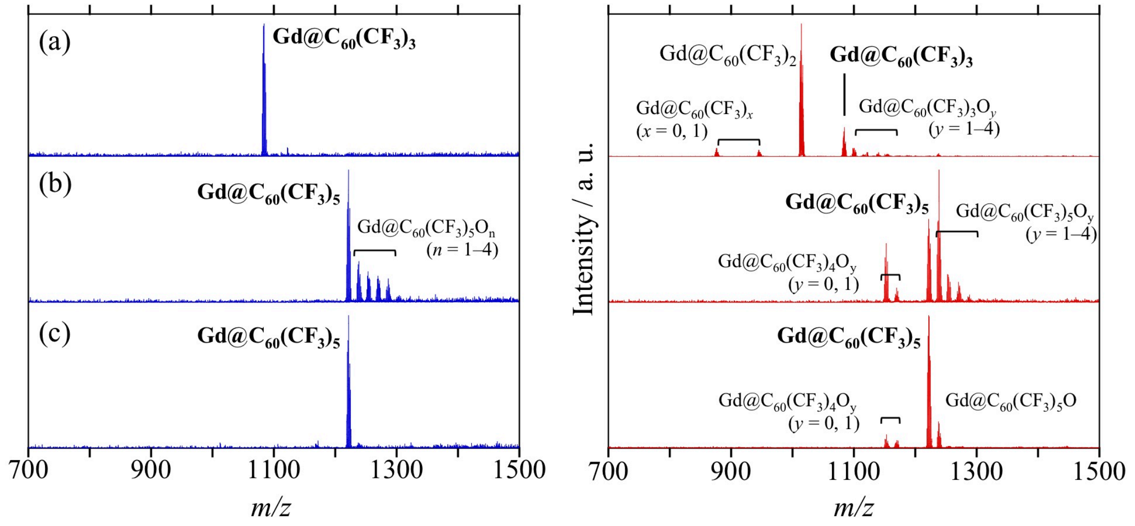

Supplementary Figure 7: MALD-TOF mass spectra with dithranol matrix (positive-ion mode in red, negative-ion mode in blue). **a**, $Gd@C_{60}(CF_3)_3$. **b**, $Gd@C_{60}(CF_3)_5$ (I). **c**, $Gd@C_{60}(CF_3)_5$ (II).

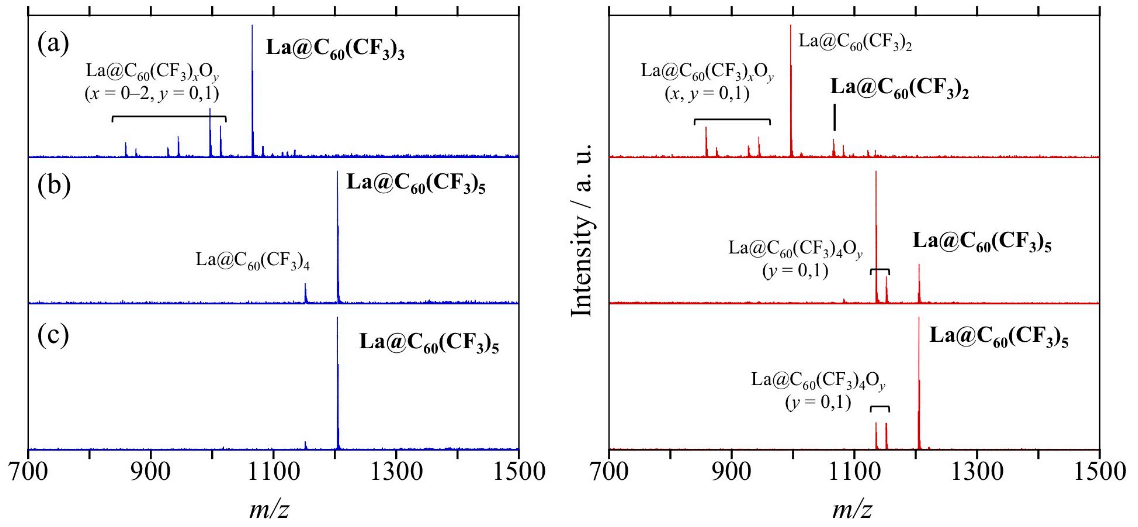

Supplementary Figure 8: MALD-TOF mass spectra with dithranol matrix (positive-ion mode in red, negative-ion mode in blue). **a**, $La@C_{60}(CF_3)_3$. **b**, $La@C_{60}(CF_3)_5$ (I). **c**, $La@C_{60}(CF_3)_5$ (II).

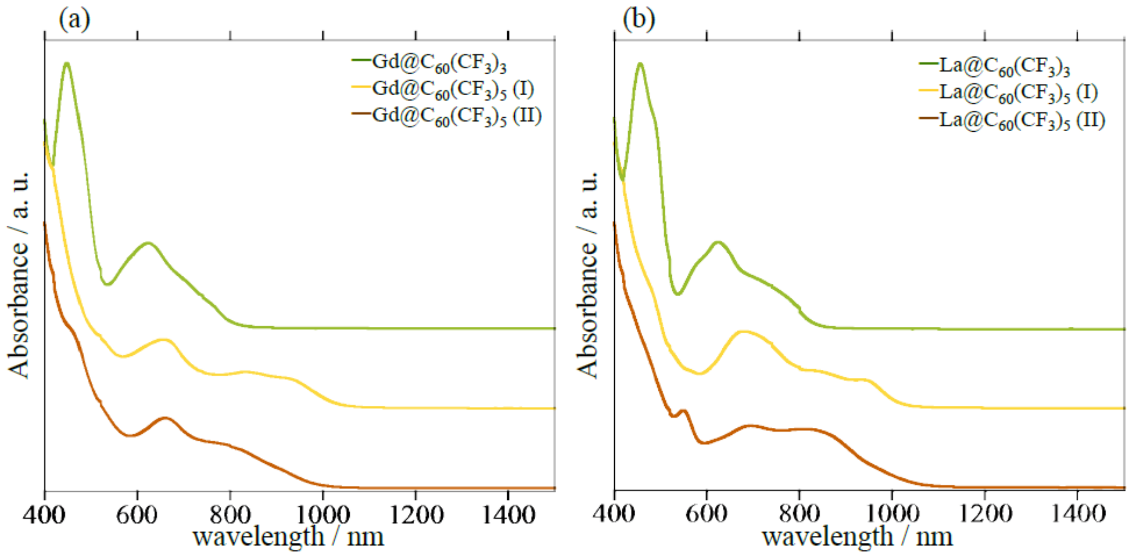

Supplementary Figure 9: UV-Vis-NIR absorption spectra in carbon disulfide for the isolated samples. **a**, $Gd@C_{60}(CF_3)_3$, $Gd@C_{60}(CF_3)_5$ (I), and $Gd@C_{60}(CF_3)_5$ (II). **b**, $La@C_{60}(CF_3)_3$, $La@C_{60}(CF_3)_5$ (I), and $La@C_{60}(CF_3)_5$ (II).

Supplementary Discussion 2: Synchrotron X-ray structure analysis

Supplementary Table 1: Crystallographic data for Gd@$C_{60}(CF_3)_5$ (I) and La@$C_{60}(CF_3)_5$ (I).

Compound	Gd@$C_{60}(CF_3)_5$ (I)	La@$C_{60}(CF_3)_5$ (I)
Formula	$GdC_{65}F_{15}$	$LaC_{65}F_{15}$
formula weight	1222.90	1204.56
crystal size (mm)	0.20 × 0.05 × 0.01	0.04 × 0.04 × 0.02
Temperature	100 K	100 K
X-ray wavelength	0.4953 Å	0.6227 Å
crystal system	monoclinic	monoclinic
space group	$P2_1/c$	$P2_1/c$
unit cell parameters	a = 10.0704(10) Å	a = 10.0725(9) Å
	b = 18.1705(19) Å	b = 18.1447(17) Å
	c = 19.554(2) Å	c = 19.6302(18) Å
	β = 96.317(7)°	β = 95.878(7)°
	V = 3556.3(6) Å^3	V = 3568.8(6) Å^3
Z	4	4
No. of independent reflections	7,256 (d > 0.80 Å)	8,859 (d > 0.75 Å)
$\Sigma\sigma_I/\Sigma I$	0.0957	0.0727
No. of parameters	676	720
$R1$	0.0595 ($\lvert F\rvert > 4\sigma$)	0.0566 ($\lvert F\rvert > 4\sigma$)
wR	0.1170 ($\lvert F\rvert > 4\sigma$)	0.1230 ($\lvert F\rvert > 4\sigma$)
GOF	1.056	1.077

Supplementary Table 2: C−C and C−F bond lengths (Å) of Gd@$C_{60}(CF_3)_5$ (I). C−C bonds around five carbon atoms with CF_3 attached (6, 9, 12, 15, and 53) are shown in boldface.

6 : 6 C−C		6 : 5 C−C		6 : 5 C−C		C−CF_3	
**1−9**	**1.500(12)**	1−2	1.401(13)	35−36	1.409(15)	**6−61**	**1.517(12)**
**2−12**	**1.505(15)**	2−3	1.387(13)	37−17	1.486(14)	**9−62**	**1.532(13)**
**3−15**	**1.504(12)**	3−4	1.437(13)	37−38	1.402(14)	**12−63**	**1.505(14)**
4−18	1.457(13)	4−5	1.438(12)	38−19	1.482(12)	**15−64**	**1.520(12)**
**5−6**	**1.508(12)**	5−1	1.415(13)	39−40	1.451(13)	**53−65**	**1.496(14)**
7−8	1.347(12)	**6−7**	**1.564(11)**	40−41	1.450(13)		
10−11	1.402(14)	**8−9**	**1.515(13)**	42−23	1.446(12)	C−F	
13−14	1.344(14)	**9−10**	**1.532(13)**	42−43	1.397(13)	61−F1	1.321(10)
16−17	1.390(14)	**11−12**	**1.526(14)**	43−44	1.454(14)	61−F2	1.358(11)
19−20	1.417(12)	**12−13**	**1.562(14)**	44−24	1.468(13)	61−F3	1.294(10)
22−23	1.410(12)	**14−15**	**1.541(13)**	45−27	1.423(14)	62−F4	1.313(11)
24−25	1.377(14)	**15−16**	**1.551(15)**	45−46	1.477(14)	62−F5	1.318(12)
26−27	1.434(13)	17−18	1.469(12)	46−47	1.383(14)	62−F6	1.316(14)
28−29	1.353(14)	18−19	1.407(14)	47−28	1.510(14)	63−F7	1.327(13)
30−31	1.398(13)	**20−6**	**1.537(12)**	48−31	1.438(12)	63−F8	1.377(12)
32−33	1.413(13)	20−21	1.460(12)	48−49	1.451(14)	63−F9	1.318(13)
34−35	1.413(14)	21−22	1.417(13)	49−50	1.486(13)	64−F10	1.343(14)
36−37	1.380(13)	22−7	1.426(13)	50−32	1.425(14)	64−F11	1.310(11)
38−39	1.426(13)	23−24	1.414(14)	51−35	1.430(13)	64−F12	1.320(13)
40−21	1.358(14)	25−8	1.435(12)	51−52	1.428(15)	65−F13	1.325(13)
41−42	1.394(13)	25−26	1.446(14)	**52−53**	**1.542(15)**	65−F14	1.343(12)
44−45	1.352(14)	26−10	1.407(14)	**53−36**	**1.578(14)**	65−F15	1.345(14)
47−48	1.383(13)	27−28	1.436(14)	54−39	1.410(13)		
50−51	1.358(15)	29−11	1.424(13)	54−55	1.425(13)	C−Gd	
**53−54**	**1.474(14)**	29−30	1.444(15)	55−41	1.452(13)	4−Gd	2.387(8)
56−55	1.390(13)	30−13	1.448(13)	56−57	1.413(14)	17−Gd	2.472(9)
57−43	1.409(13)	31−32	1.419(14)	57−58	1.435(15)	18−Gd	2.351(8)
58−46	1.387(15)	33−14	1.432(13)	58−59	1.475(14)	19−Gd	2.375(9)
59−49	1.375(14)	33−34	1.424(16)	59−60	1.439(15)		
60−52	1.359(13)	34−16	1.402(13)	60−56	1.470(14)		

Supplementary Table 3: C–C and C–F bond lengths (Å) of La@$C_{60}(CF_3)_5$ (I). C–C bonds around five carbon atoms with CF_3 attached (6, 9, 12, 15, and 53) are shown in boldface.

6 : 6 C–C		6 : 5 C–C		6 : 5 C–C		C–CF_3	
**1–9**	**1.508(7)**	1–2	1.402(7)	35–36	1.422(9)	**6–61**	**1.527(7)**
**2–12**	**1.499(7)**	2–3	1.417(8)	37–17	1.457(8)	**9–62**	**1.530(7)**
**3–15**	**1.513(7)**	3–4	1.424(7)	37–38	1.438(8)	**12–63**	**1.526(8)**
4–18	1.463(7)	4–5	1.438(6)	38–19	1.459(7)	**15–64**	**1.519(7)**
**5–6**	**1.509(7)**	5–1	1.405(7)	39–40	1.450(7)	**53–65**	**1.523(9)**
7–8	1.377(7)	**6–7**	**1.548(6)**	40–41	1.436(7)		
10–11	1.361(8)	**8–9**	**1.531(7)**	42–23	1.449(7)	C–F	
13–14	1.345(9)	**9–10**	**1.547(7)**	42–43	1.434(7)	61–F1	1.327(6)
16–17	1.409(8)	**11–12**	**1.545(8)**	43–44	1.444(7)	61–F2	1.336(6)
19–20	1.417(7)	**12–13**	**1.567(8)**	44–24	1.444(7)	61–F3	1.325(6)
22–23	1.393(7)	**14–15**	**1.560(8)**	45–27	1.451(8)	62–F4	1.307(6)
24–25	1.388(7)	**15–16**	**1.522(8)**	45–46	1.443(8)	62–F5	1.331(7)
26–27	1.405(7)	17–18	1.463(7)	46–47	1.449(8)	62–F6	1.293(7)
28–29	1.380(8)	18–19	1.441(7)	47–28	1.473(8)	63–F7	1.339(7)
30–31	1.408(8)	**20–6**	**1.554(7)**	48–31	1.444(8)	63–F8	1.335(7)
32–33	1.401(8)	20–21	1.422(7)	48–49	1.428(8)	63–F9	1.324(7)
34–35	1.412(8)	21–22	1.454(7)	49–50	1.440(8)	64–F10	1.345(8)
36–37	1.398(15)	22–7	1.435(7)	50–32	1.471(8)	64–F11	1.337(6)
38–39	1.425(7)	23–24	1.427(7)	51–35	1.447(8)	64–F12	1.321(8)
40–21	1.394(7)	25–8	1.430(7)	51–52	1.421(8)	65–F13	1.344(8)
41–42	1.394(7)	25–26	1.441(7)	**52–53**	**1.535(9)**	65–F14	1.330(8)
44–45	1.390(7)	26–10	1.424(7)	**53–36**	**1.553(8)**	65–F15	1.347(9)
47–48	1.376(8)	27–28	1.430(8)	54–39	1.406(8)		
50–51	1.385(9)	29–11	1.442(7)	54–55	1.440(7)	C–La	
**53–54**	**1.497(8)**	29–30	1.446(8)	55–41	1.447(7)	4–La	2.519(4)
56–55	1.386(8)	30–13	1.433(8)	56–57	1.448(8)	17–La	2.593(5)
57–43	1.386(7)	31–32	1.416(9)	57–58	1.459(8)	18–La	2.498(5)
58–46	1.390(8)	33–14	1.429(8)	58–59	1.440(8)	19–La	2.518(5)
59–49	1.424(8)	33–34	1.440(9)	59–60	1.420(9)		
60–52	1.401(8)	34–16	1.419(7)	60–56	1.458(8)		

Supplementary Table 4: Crystallographic data for $Gd@C_{60}(CF_3)_5$ (II).

Compound	$Gd@C_{60}(CF_3)_5$ (II)
formula	$GdC_{65}F_{15}\cdot(NiC_{36}N_4H_{44})_{1.5}\cdot(C_7H_8)_{0.23}$
formula weight	2131.67
crystal size (mm)	0.04 × 0.04 × 0.01
temperature	100 K
X-ray wavelength	0.7022 Å
crystal system	monoclinic
space group	$P-1$
unit cell parameters	a = 14.458(4) Å
	b = 14.662(4) Å
	c = 21.393(7) Å
	α = 84.799(6)°
	β = 86.774(8)°
	γ = 72.339(6)°
	V = 4302(2) Å^3
Z	2
No. of independent reflections	16,856 (d > 0.80 Å)
$\Sigma\sigma_I/\Sigma I$	0.0942
No. of parameters	1,487
$R1$	0.0573 ($\lvert F\rvert > 4\sigma$)
wR	0.1283 ($\lvert F\rvert > 4\sigma$)
GOF	0.982

Supplementary Table 5: C−C and C−F bond lengths (Å) of $Gd@C_{60}(CF_3)_5$ (II). C−C bonds around five carbon atoms with CF_3 attached (6, 9, 12, 15, and 36) are shown in boldface.

6 : 6 C−C		6 : 5 C−C		6 : 5 C−C		C−CF_3	
**1−9**	**1.521(11)**	1−2	1.393(8)	**35−36**	**1.526(9)**	**6−61**	**1.520(12)**
**2−12**	**1.525(11)**	2−3	1.417(11)	37−17	1.464(10)	**9−62**	**1.522(11)**
**3−15**	**1.510(12)**	3−4	1.456(11)	37−38	1.417(10)	**12−63**	**1.545(10)**
4−18	1.469(8)	4−5	1.432(12)	38−19	1.481(11)	**15−64**	**1.546(10)**
**5−6**	**1.515(12)**	5−1	1.422(12)	39−40	1.424(10)	**36−65**	**1.536(9)**
7−8	1.364(11)	**6−7**	**1.562(11)**	40−41	1.450(11)		
10−11	1.361(8)	**8−9**	**1.546(10)**	42−23	1.448(11)	C−F	
13−14	1.372(9)	**9−10**	**1.545(10)**	42−43	1.456(12)	61−F1	1.372(10)
16−17	1.443(9)	**11−12**	**1.558(9)**	43−44	1.448(11)	61−F2	1.345(10)
19−20	1.377(10)	**12−13**	**1.534(11)**	44−24	1.447(12)	61−F3	1.312(11)
22−23	1.405(11)	**14−15**	**1.556(10)**	45−27	1.463(11)	62−F4	1.359(10)
24−25	1.407(11)	**15−16**	**1.552(10)**	45−46	1.446(12)	62−F5	1.329(11)
26−27	1.373(11)	17−18	1.438(10)	46−47	1.461(11)	62−F6	1.326(9)
28−29	1.407(10)	18−19	1.475(10)	47−28	1.453(12)	63−F7	1.343(10)
30−31	1.397(11)	**20−6**	**1.542(11)**	48−31	1.462(11)	63−F8	1.338(10)
32−33	1.406(10)	20−21	1.418(10)	48−49	1.453(12)	63−F9	1.297(9)
34−35	1.406(9)	21−22	1.462(10)	49−50	1.468(12)	64−F10	1.366(9)
**36−37**	**1.521(10)**	22−7	1.460(11)	50−32	1.447(11)	64−F11	1.316(11)
38−39	1.401(10)	23−24	1.439(11)	51−35	1.455(10)	64−F12	1.336(10)
40−21	1.422(11)	25−8	1.450(11)	51−52	1.456(9)	65−F13	1.339(8)
41−42	1.383(10)	25−26	1.464(11)	52−53	1.454(11)	65−F14	1.327(9)
44−45	1.393(11)	26−10	1.448(10)	**53−36**	**1.560(9)**	65−F15	1.350(8)
47−48	1.372(11)	27−28	1.437(8)	54−39	1.486(11)		
50−51	1.391(10)	29−11	1.439(11)	54−55	1.446(10)	C−Gd	
53−54	1.365(10)	29−30	1.445(10)	55−41	1.416(11)	4−Gd	2.390(11)
56−55	1.409(12)	30−13	1.436(10)	56−57	1.462(12)	17−Gd	2.390(8)
57−43	1.374(12)	31−32	1.440(10)	57−58	1.466(15)	18−Gd	2.377(8)
58−46	1.401(9)	33−14	1.450(10)	58−59	1.423(14)	19−Gd	2.531(8)
59−49	1.410(12)	33−34	1.460(9)	59−60	1.460(13)		
60−52	1.393(11)	34−16	1.455(10)	60−56	1.432(10)		

Supplementary Table 6: Crystallographic data for Gd@$C_{60}(CF_3)_3$.

Compound	Gd@$C_{60}(CF_3)_3$
formula	$GdC_{63}F_9$
formula weight	1084.9
crystal size (mm)	0.10 × 0.01 × 0.01
temperature	100 K
X-ray wavelength	0.61988 Å
crystal system	tetragonal
space group	$P4_2/m$
unit cell parameters	a = 36.955(8) Å
	c = 9.886(1) Å
	V = 13501(7) Å^3
Z	16
No. of independent reflections	3,142 (d > 1.20 Å, $\|F\| > 3\sigma$)
$\Sigma\sigma_I/\Sigma I$	0.1363

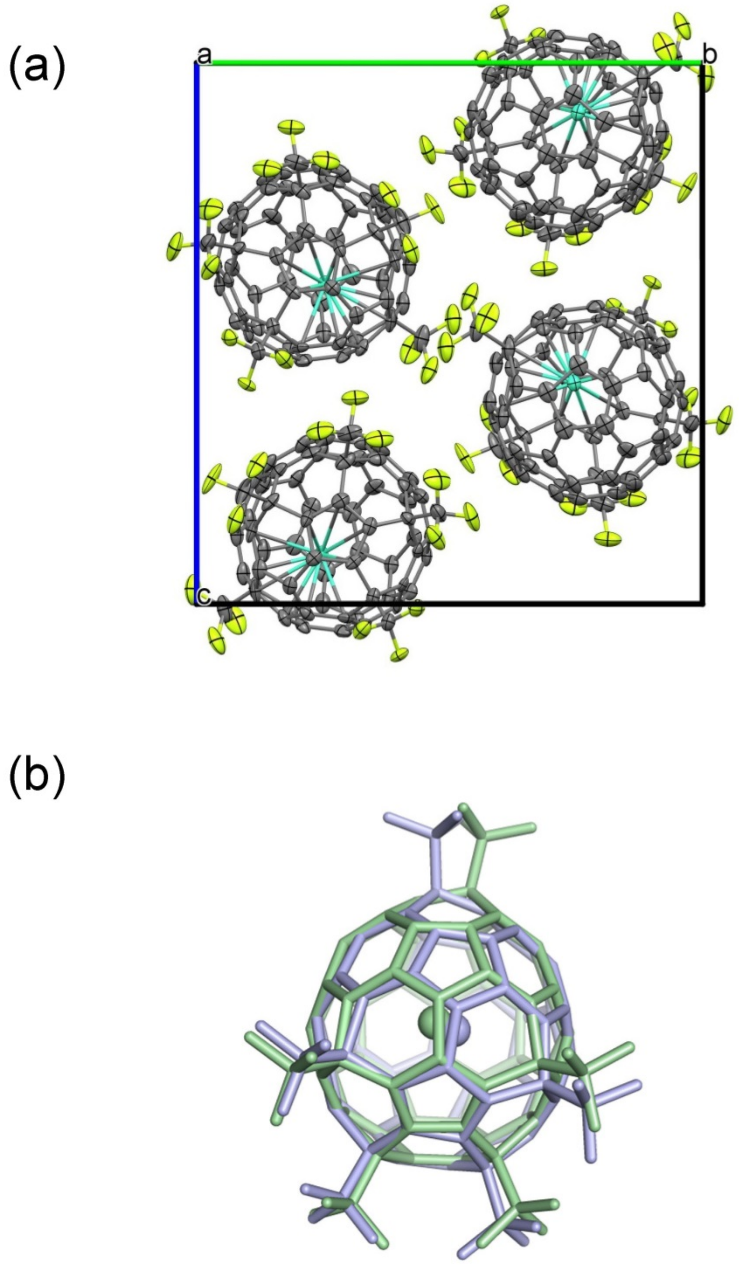

Supplementary Figure 10: **a**, Crystal structure of Gd@$C_{60}(CF_3)_5$ (I) viewed along the *a*-axis. The thermal ellipsoids are drawn at 50% probability level. **B**, Overlapped two chiral isomers, the major light green and the minor light blue isomers. The molecular disorder is omitted in **a**.

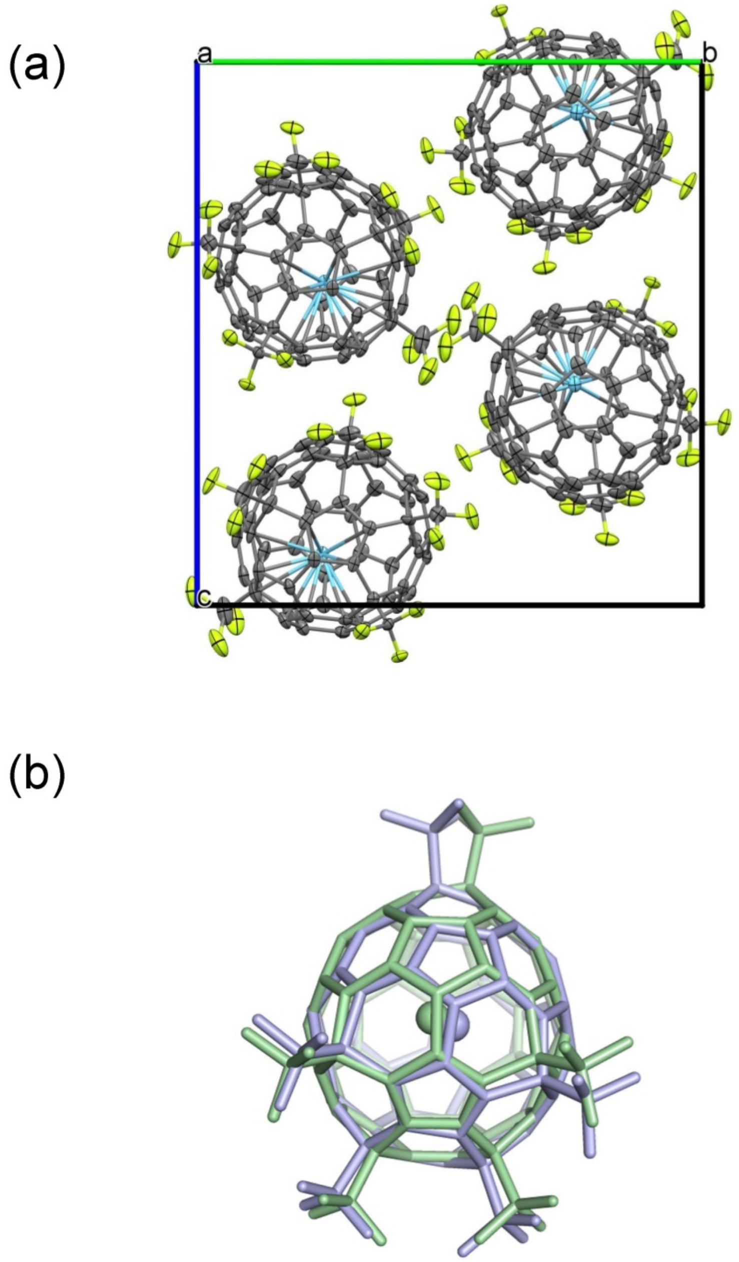

Supplementary Figure 11: **a**, Crystal structure of La@$C_{60}(CF_3)_5$ (I) viewed along the *a*-axis. The thermal ellipsoids are drawn at 50% probability level. **B**, Overlapped two chiral isomers, the major light green and the minor light blue isomers. The molecular disorder is omitted in **a**.

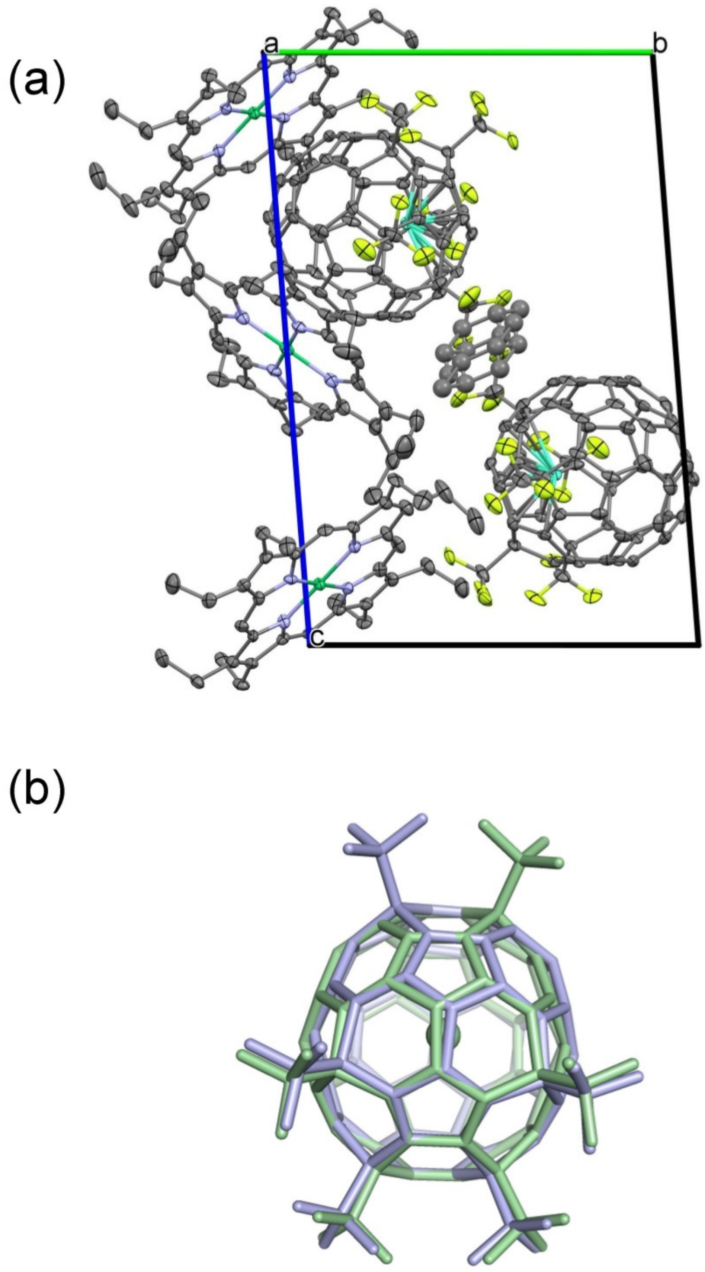

Supplementary Figure 12: a, Crystal structure of $Gd@C_{60}(CF_3)_5$ (II) viewed along the *a*-axis. The thermal ellipsoids are drawn at 50% probability level. **B**, Overlapped two chiral isomers, the major light green and the minor light blue isomers. The molecular disorder and hydrogen atoms are omitted in **a**.

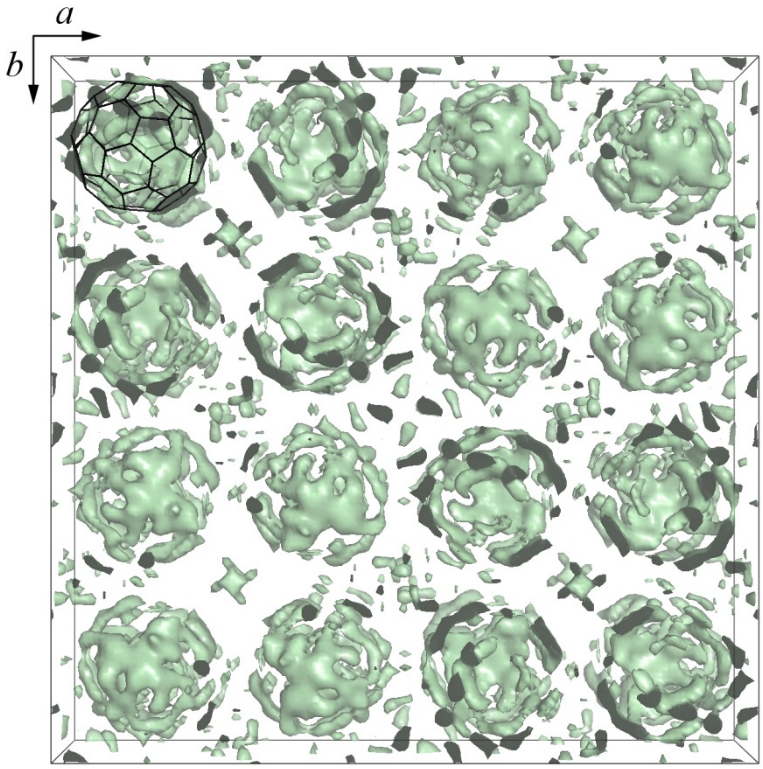

Supplementary Figure 13: Charge density surface of $Gd@C_{60}(CF_3)_3$ obtained by maximum entropy method assuming an expected molecular arrangement.

Supplementary Discussion 3: ^{19}F-NMR measurements

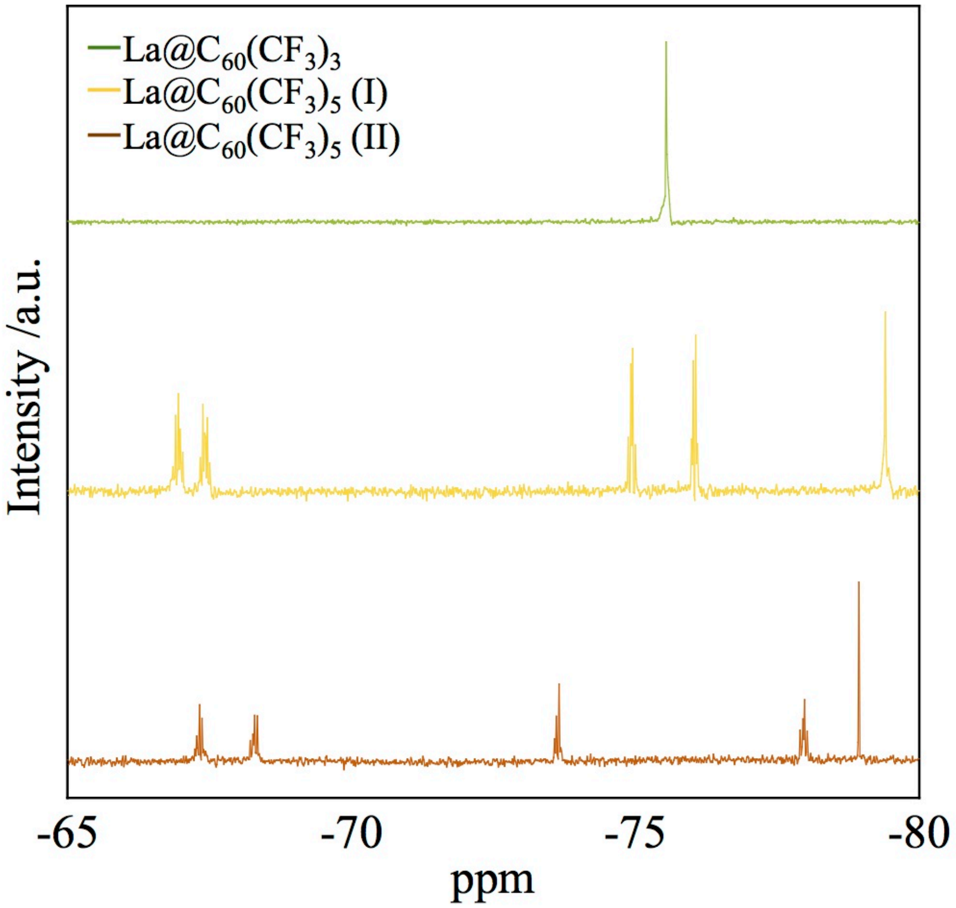

Supplementary Figure 14: ^{19}F NMR spectra of La@$C_{60}(CF_3)_3$, La@$C_{60}(CF_3)_5$ (I), and La@$C_{60}(CF_3)_5$ (II).

Supplementary Discussion 4: Theoretical calculations

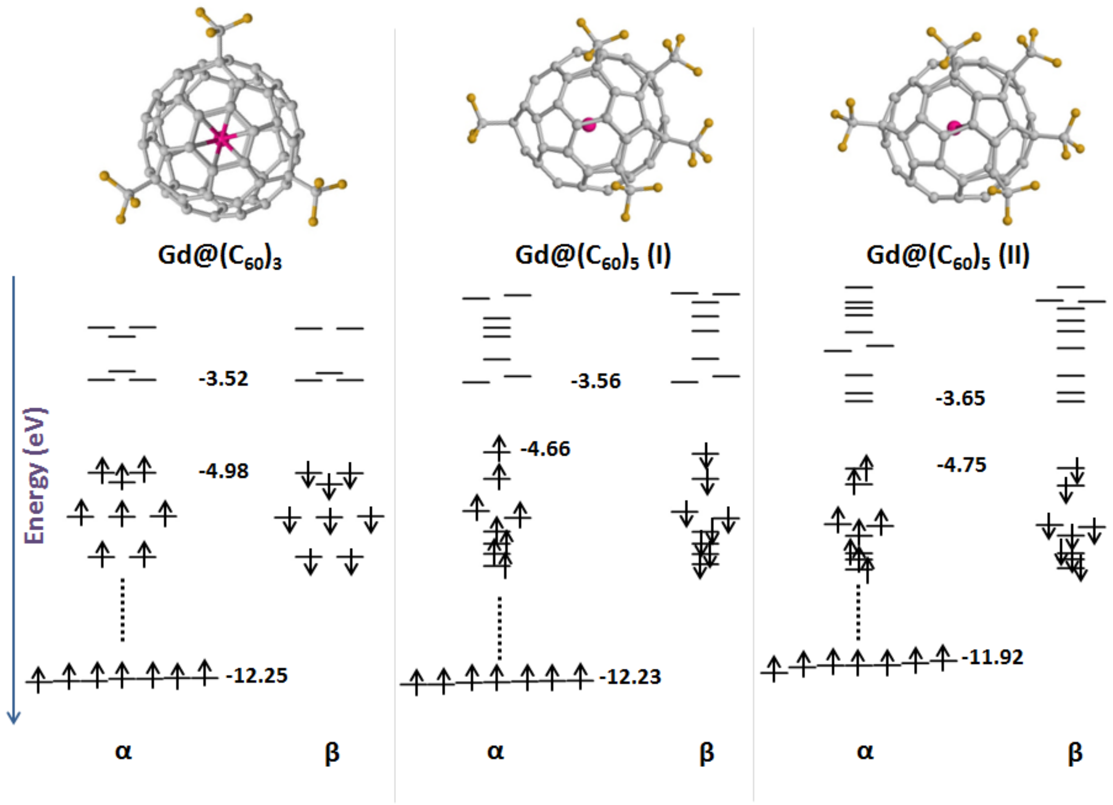

Supplementary Figure 15: Calculated Kohn-Sham eigenvalues (eV) for (left) Gd@$C_{60}(CF_3)_3$, (centre) Gd@$C_{60}(CF_3)_5$ (I), and (right) Gd@$C_{60}(CF_3)_5$ (II), showing CF_3 functionalisation results in a closed shell system, re-opening the HOMO-LUMO gap to nearly that of C_{60}. Of the three species, the calculated gap is largest for Gd@$C_{60}(CF_3)_3$ (calculated HOMO-LUMO gap of C_{60}=1.67eV, Gd@$C_{60}(CF_3)_3$=1.46eV, Gd@$C_{60}(CF_3)_5$ (I and II)=1.10eV). For comparison, an unstable alternative Gd@$C_{60}(CF_3)_5$ isomer with all five pentagonal back bonds functionalised with CF_3 has a calculated gap of only 0.28eV. Note that these systems were also re-optimised with PBE-GGA for comparison, giving almost identical structures and HOMO-LUMO gaps.

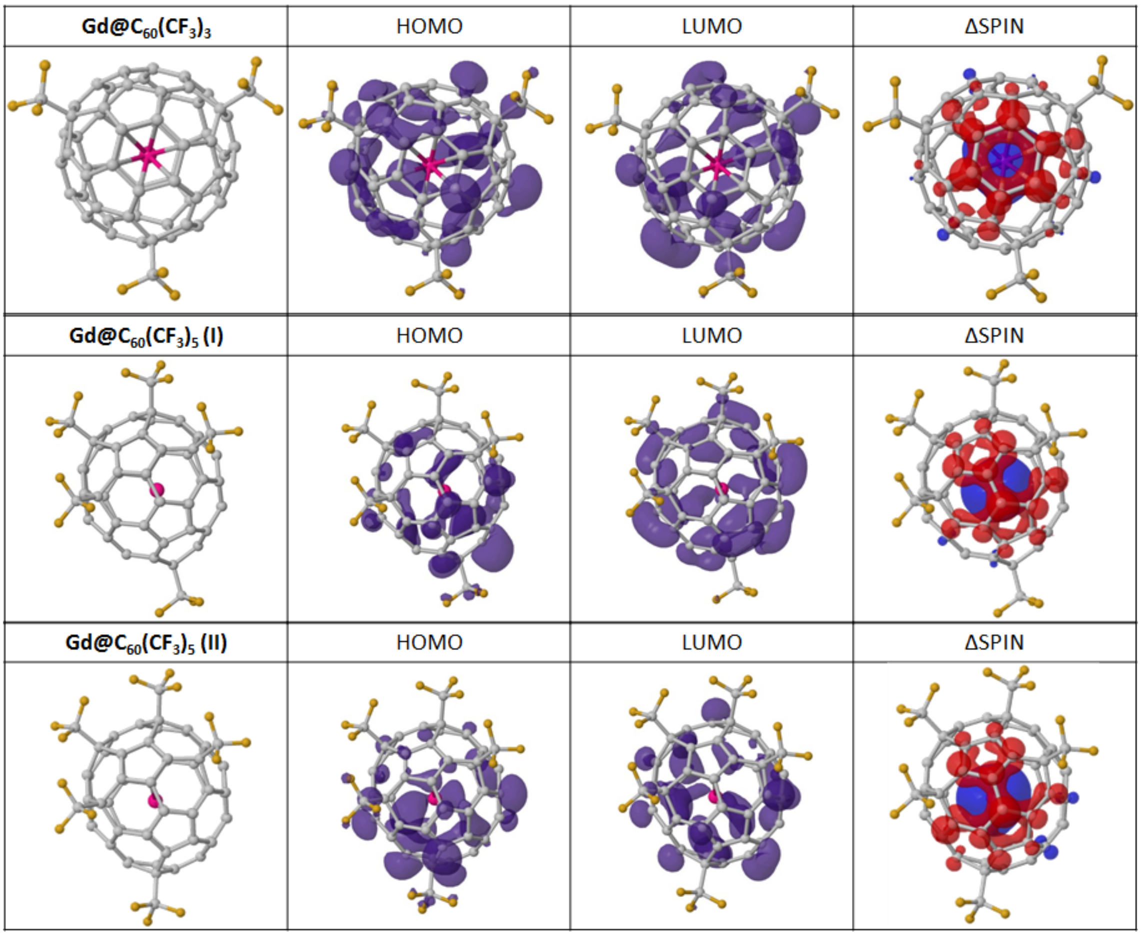

Supplementary Figure 16: Spatial distribution of calculated Kohn-Sham eigenstates showing highest occupied molecular orbital (HOMO) and lowest unoccupied molecular orbital (LUMO), and difference in spin-up and spin-down charge density for $Gd@C_{60}(CF3)_3$, $Gd@C_{60}(CF3)_5$ (I) and $Gd@C_{60}(CF3)_5$ (II). The HOMO and LUMO states are delocalised across the fullerene cage with very limited CF_3 character. The spin density distribution shows a large maximum centred on the Gd, with weak counter-spin localised on fullerene cage carbon atoms neighbouring the Gd.

Supplementary Discussion 5: SQUID magnetic measurement

The temperature dependence of molar magnetic susceptibility χ_m of Gd@$C_{60}(CF_3)_3$ is shown in Supplementary Figure 17a, which is well fitted by the Curie-Weiss law represented by

$$\chi_{\rm m} = \chi_{\rm c} + \frac{Ng^2J(J+1)\mu_{\rm B}^2}{3k_{\rm B}(T-\Theta)} = \chi_{\rm c} + \frac{N\mu_{\rm eff}^2}{3k_{\rm B}(T-\Theta)}$$

with $J = 7/2$, Θ = -0.14 and $N = 6.34\times10^{23}$ mol^{-1} (almost the same as Avogadro's number), where χ_c is a temperature independent constant term and Θ the Weiss temperature. This also supports a $\mu_{\rm eff}$ of 7.94μ_B for each Gd@$C_{60}(CF_3)_3$ molecule. Inset of Supplementary Figure 17a indicates that the extrapolation of a slope of inverse magnetic susceptibility against temperature crosses at a temperature of -0.14 K, which suggests that a very small antiferromagnetic coupling exists between the magnetic moment on gadolinium ion and a trace counter-spin 24ocalized on the C_{60} cage /or between the magnetic moments on gadolinium ions. It is thus reasonable to observe a small decrease of J at 2 K as seen in the bottom panel of Figure 3. From the analyses of magnetization curves and temperature dependence of χ_m, we obtain $J = 7/2$ and $N = 6.34\times10^{23}$ mol^{-1}. Using these parameters, we obtained an expected value for M_s (= $NgJ\mu_B$) of 41,140 emu·G/mol, in good agreement with M_s of 41,108 emu·G/mol obtained from the magnetization curve measurements. However, if each Gd@$C_{60}(CF_3)_3$ molecule has $J = 7/2$, N should be equal to N_A, giving an ideal value for M_s (= $N_A\, g\, J\, \mu_B$) of 39,064 emu·G/mol. We think that the difference between this ideal value and experimental M_s originates in the measurement error of the sample weight loaded in the SQUID quartz tube, *i.e.*, an inaccuracy of ~0.04 mg with respect to 0.71 mg. The magnetization curve of La@$C_{60}(CF_3)_5$ solid shows a linear response with respect to the magnetic field down to 2 K, suggesting a small $\mu_{\rm eff}$ if it exists. The temperature dependence of χ_m behaves Curie-like as shown in Supplementary Figure 17b, and is roughly fitted by

$$\chi_{\rm m} = \chi_{\rm c} + \frac{N_{\rm A}\mu_{\rm eff}^2}{3k_{\rm B}T}$$

with $\mu_{\rm eff}$ of 0.35μ_B per La@$C_{60}(CF_3)_5$ molecule, where N_A is Avogadro's number. Hence, 3 electrons of encaged La are transferred to the cage (La^{3+}@C_{60}^{3-}) giving La^{3+} ($5p^6$, $J = 0$) and these electrons are distributed to five CF_3 in order to make chemical bonds with C_{60}. This may cause a small $\mu_{\rm eff}$ on the cage due to the difference between α and β spin concentrations. The $\mu_{\rm eff}$ of La@$C_{60}(CF_3)_5$ is almost the same with that of La@C_{82}[5]. The valency of La of La@C_{82} is also 3+, and the transferred electrons to the C_{82} cage are uniformly diffused on the cage. We therefore think that a similar magnetic feature was observed for the present La@C_{60} fulleride.

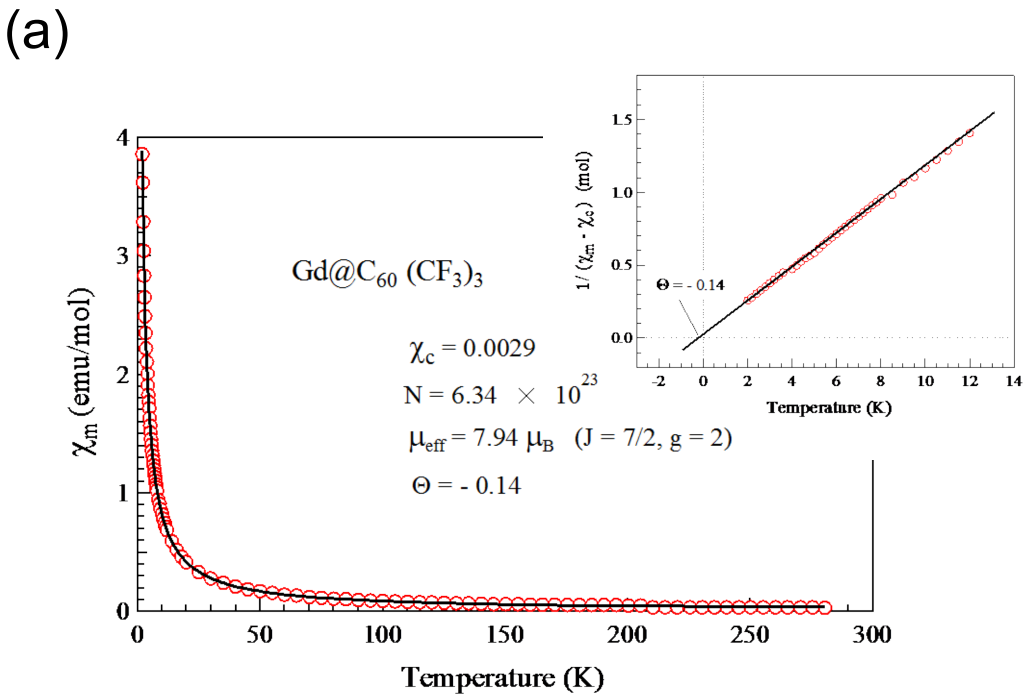

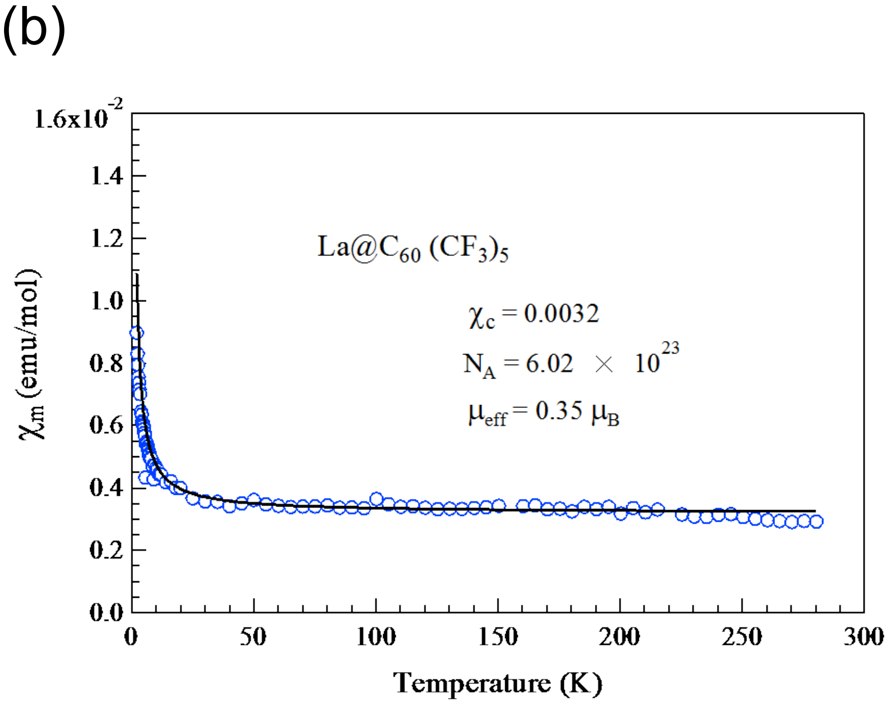

Supplementary Figure 17: a, Temperature dependence of molar magnetic susceptibility χ_m for Gd@$C_{60}(CF_3)_3$. Inset indicates the inverse of temperature dependent term of χ_m with respect to temperature. **B**, Temperature dependence of χ_m for La@$C_{60}(CF_3)_5$.

Supplementary Discussion 6: Electron spin resonance (ESR) measurements

Supplementary Table 7: Spin Hamiltonian Parameters for Gd@$C_{60}(CF_3)_5$ (I) and Gd@$C_{60}(CF_3)_5$ (II) in CS_2.

	isomer I	isomer II
S	7/2	7/2
g	(2.008 2.008 2.008)	(1.987 2.003 1.987)
D / cm^{-1}	0.1617	0.1934
E / cm^{-1}	0.0000	0.01241

References

1. Akiyama, K. *et al*. Non-HPLC rapid separation of metallofullerenes and empty cages with $TiCl_4$ Lewis acid. *J. Am. Chem. Soc.* **134**, 9762-9767 (2012).
2. Wang, Z., Nakanishi, Y., Noda, S., Akiyama, K. & Shinohara, H. The origin and mechanism of non-HPLC purification of metallofullerenes with $TiCl_4$. *J.Phys.Chem.C*, **116**, 25563-25567 (2012).
3. Shinohara, H. Endohedral metallofullerenes. *Rep. Prog. Phys*. **63**, 843-892 (2000).
4. Shinohara, H. & Tagmatarchis, N. *Endohedral Metallofullerenes - Fullerenes with Metal Inside* - (Wiley, 2015).
5. Funasaka, H, Sugiyama. K., Yamamoto, K. & Takahashi, T. Magnetic Properties of rare-earth metallofullerenes. *J. Phys. Chem.* **99**, 1826-1830 (1995).
6. Stoll, S. & Schweiger, A. Easy Spin, a comprehensive software package for spectral simulation and analysis in EPR. *J. Magn. Reson.* **178**, 42-55 (2006).
7. http://www.easyspin.org/
